# TRAF6 maintains mammary stem cells and promotes pregnancy-induced mammary epithelial cell expansion

**DOI:** 10.1038/s42003-019-0547-7

**Published:** 2019-08-06

**Authors:** Mizuki Yamamoto, Chiho Abe, Sakura Wakinaga, Kota Sakane, Yo Yumiketa, Yuu Taguchi, Takayuki Matsumura, Kosuke Ishikawa, Jiro Fujimoto, Kentaro Semba, Maki Miyauchi, Taishin Akiyama, Jun-ichiro Inoue

**Affiliations:** 1 Division of Cellular and Molecular Biology, The Institute of Medical Science, The University of Tokyo, Shirokane-dai, Minato-ku, Tokyo 108-8639 Japan; 2 Research Center for Asian Infectious Diseases, The Institute of Medical Science, The University of Tokyo, Shirokane-dai, Minato-ku, Tokyo 108-8639 Japan; 3 Department of Immunology, National Institute of Infectious Diseases, Toyama, Shinjuku-ku, Tokyo 162-8640 Japan; 4 Department of Life Science and Medical Bio-Science, Waseda University, Shinjuku-ku, Tokyo 162-8480 Japan; 5 Laboratory for Immune Homeostasis, RIKEN Center for Integrative Medical Sciences, Tsurumi-ku, Yokohama, Kanagawa 230-0045 Japan

**Keywords:** Cell proliferation, Organogenesis, Cell growth

## Abstract

Receptor activator of nuclear factor (NF)-κB (RANK) signaling promotes pregnancy-dependent epithelial cell differentiation and expansion for mammary gland development, which requires NF-κB pathway-dependent Cyclin D1 induction and inhibitor of DNA binding 2 (Id2) pathway-dependent anti-apoptotic gene induction. However, the roles of tumor necrosis factor receptor-associated factor 6 (TRAF6) remain unclear despite its requirement in RANK signaling. Here we show that TRAF6 is crucial for both mammary stem cell maintenance and pregnancy-induced epithelial cell expansion. TRAF6 deficiency impairs phosphoinositide 3-kinase (PI3K)/AKT and canonical NF-κB pathways, whereas noncanonical NF-κB signaling remains functional. Therefore, we propose that TRAF6 promotes cell proliferation by activating PI3K/AKT signaling to induce retinoblastoma phosphorylation in concert with noncanonical NF-κB pathway-dependent Cyclin D1 induction. Furthermore, TRAF6 inhibits apoptosis by activating canonical NF-κB signaling to induce anti-apoptotic genes with the Id2 pathway. Therefore, proper orchestration of TRAF6-dependent and -independent RANK signals likely establishes mammary gland formation.

## Abstract (web summary)

Mizuki Yamamoto et al. show that TRAF6 is needed for mammary stem cell maintenance and pregnancy-induced epithelial cell expansion in cells and mice. They propose a model whereby TRAF6 promotes cell proliferation via PI3K/AKT signalling and inhibits apoptosis by activating canonical NF-κB signalling.

## Introduction

Mammary glands are unique organs that produce milk to feed neonatal offspring for alimentation and passive immunity^1^. The mammary epithelium is mainly composed of two types of cellular lineages differentiated from mammary stem cells (MaSCs)^2,3^. These cell types are luminal epithelial cells, which constitute the inner layer, surrounding a central lumen and basal epithelial (myoepithelial) cells, which constitute the outer layer, bordering the basal lamina. Reciprocal signaling between the epithelium and the mesenchyme during embryonic development leads to mammary anlage formation^4^. During puberty, tubules form from the epithelium by elongation and bifurcation, establishing the basic arboreal networks emanating from the nipples^5^. Cell proliferation at the tips of the ducts leads to the formation of spoon-shaped terminal end buds. During pregnancy, the ductal cells rapidly proliferate to form alveolar structures. Luminal cells then undergo maturation to express milk-related genes^6^. During lactation, contraction of the basal cells causes the milk to be ejected through the ducts into the nipple for the suckling infant. Following weaning, the mammary gland undergoes rapid involution characterized by massive epithelial cell death^7^. These sequential processes involve generation and maintenance of MaSCs and their commitment to specific differentiation pathways, proliferation of committed progenitor cells, execution of differentiation programs, and the maintenance of tissue homeostasis by cell survival control. These complex programs are tightly regulated by various signaling pathways triggered by critical ligands including steroid and peptide hormones and cytokines^8^.

Among these ligands, receptor activator of nuclear factor (NF)-κB ligand (RANKL) is a cytokine whose deficiency results in a complete blockade of mammary gland development during pregnancy^9^. RANKL is a member of the tumor necrosis factor (TNF) superfamily (TNFSF) and binds to its receptor RANK, a member of the TNF receptor (TNFR) superfamily, whose deficiency also suppresses the rapid, pregnancy-induced proliferation of ductal cells, alveolar formation, and milk production without any morphological defects in the mammary gland during puberty. Moreover, RANK signaling is involved in the maintenance of the MaSC population in virgin mice^10^.

Two distinct NF-κB activation pathways have been identified downstream of RANK^11,12^. The canonical pathway activates the inhibitor of NF-κB (IκB) kinase (IKK) complex, which is composed of the catalytic subunits IKKα and IKKβ, and the regulatory subunit NF-κB essential modulator (NEMO, IKKγ)^13^. Then, the IKK complex induces the phosphorylation and subsequent proteasome-dependent degradation of IκBα, which allows the p50/RelA NF-κB heterodimer to translocate into the nucleus and activate target genes. In contrast, the noncanonical pathway activates IKKα without IKKβ and NEMO, in an NF-κB-inducing kinase (NIK)-dependent manner. IKKα then phosphorylates the C-terminal ankyrin repeats of p100, which form heterodimers with RelB in the cytoplasm, leading to the proteasome-dependent selective degradation of the p100 C-terminal end to generate p52^14^. The resulting p52/RelB NF-κB heterodimer translocates into the nucleus and activates target genes. The canonical and noncanonical pathways have distinct physiological functions^15,16^, which strongly suggests that their target gene profiles differ^17^. It has been demonstrated that pregnancy-induced mammary epithelial cell proliferation and RANKL stimulation-dependent Cyclin D1 induction are blocked in mice carrying an inactive IKKα mutant (*Ikkα*^AA/AA^)^18^. In addition, the defective cell proliferation is rescued following exposure to a sufficient amount of Cyclin D1 expression, indicating the crucial role of RANKL-induced Cyclin D1 expression in pregnancy-induced mammary epithelial cell proliferation. As *Ikkα*^AA/AA^ cannot activate the canonical or noncanonical NF-κB pathway in RANK signaling^18^, the specific roles of each NF-κB pathway in mammary gland development have not been addressed.

TNFR-associated factor 6 (TRAF6) is an E3 ubiquitin ligase that generates Lys63-linked ubiquitin chains and is crucial for RANKL-induced NF-κB activation^19–23^, thereby promoting osteoclastogenesis, lymph node formation, and the development of medullary thymic epithelial cells^20,24–27^. In this study, we show that lack of TRAF6 selectively abrogates the canonical pathway while scarcely affecting the noncanonical pathway-mediated gene expression, thereby successfully distinguishing the roles of each NF-κB pathway in RANK signaling-mediated mammary gland development. In combination with the results from other extensive signal analyses, we propose a novel and precise model of RANK signal pathways that cooperatively promote pregnancy-induced mammary gland development.

## Results

### TRAF6 is unneeded for mammary gland development until virgin

**Fig. 1:**
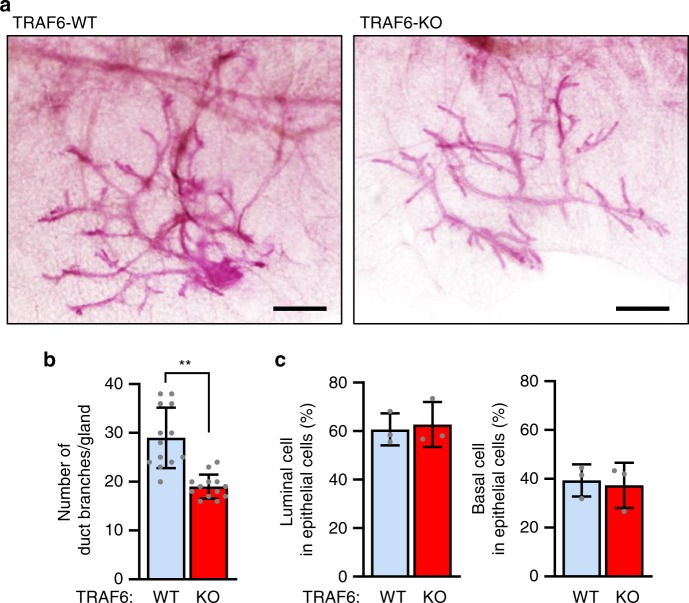
TRAF6 is partially involved in branching of mammary ducts but not essential for mammary gland development during prepuberty. **a** Whole-mount analysis of mammary tissue of 10-day-old TRAF6-WT and TRAF6-KO female mice. Scale bars, 500 μm. **b** Branching morphogenesis in mammary tissue of 10-day-old TRAF6-WT and TRAF6-KO mice. Values are means ± SD (TRAF6-WT, *n* = 13 and TRAF6-KO, *n* = 13; **p < 0.01). **c** Populations of CD24^high^CD49f^mid^ luminal cells (left) and CD24^mid^CD49f^high^ basal cells (right) were analyzed using flow cytometry. Mammary epithelial cells isolated from 10-day-old female TRAF6-WT and TRAF6-KO mice were stained for CD24 and CD49f expression. Values are means ± SD (TRAF6-WT and TRAF6-KO, *n* = 3/group)

To investigate the role of TRAF6 in prepubertal mammary gland development, a whole-mount analysis of the mammary glands of 10-day-old female TRAF6 wild-type (WT) and -knockout (KO; *Traf6*^+/+^ and *Traf6*^–/–^, respectively) mice was performed. Whereas tubule formation was nearly normal (Fig. 1a), branching morphogenesis was significantly but slightly diminished in the absence of TRAF6 (Fig. 1b and Supplementary Fig. 1). A flow cytometric analysis of the mammary epithelia of these mice showed no appreciable differences in the population sizes of CD24^high^CD49f^mid^ luminal and CD24^mid^CD49f^high^ basal cells (Fig. 1c), indicating that TRAF6 is dispensable for prepubertal differentiation of mammary epithelia.

**Fig. 2:**
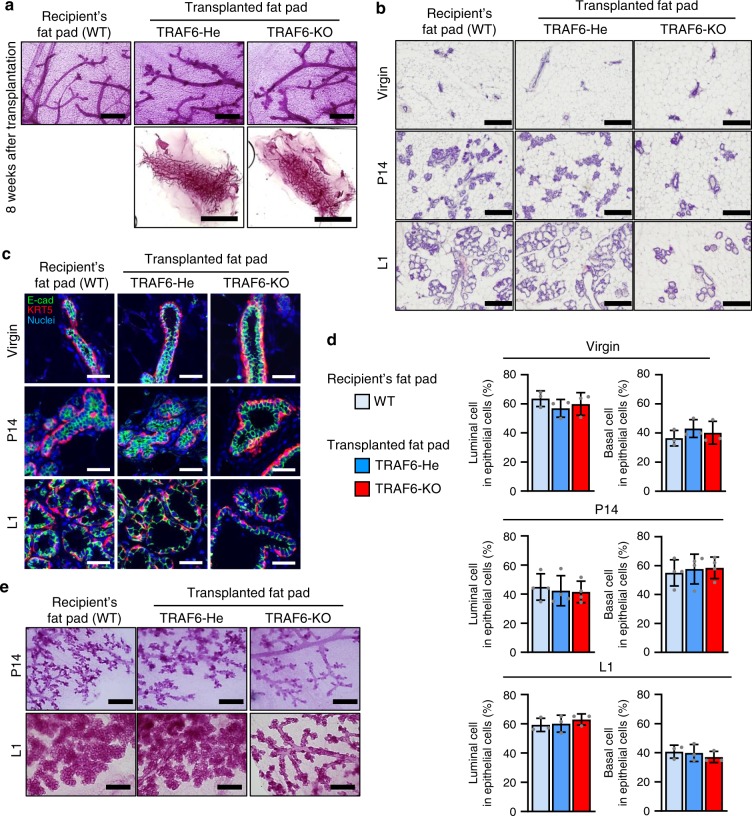
TRAF6 is essential for pregnancy-induced alveologenesis. **a** Whole-mount analysis of the mammary gland in #2 fat pads of recipient wild-type mice and the outgrowths derived from transplanted TRAF6-He and TRAF6-KO epithelia in cleared fat pads at 8 weeks after transplantation. Scale bars, 500 μm (upper panels) or 1 cm (lower panels). **b** Hematoxylin and eosin (H&E) staining of the outgrowths in cleared fat pads of recipient mice. Scale bars, 200 μm. **c** Immunostaining of outgrowths in cleared fat pads of recipient mice with antibodies specific to Keratin 5 (Krt5, red) and E-cadherin (E-cad, green). Nuclei were stained with Hoechst 33342 (blue). Scale bars, 100 μm. **d** Luminal and basal cell populations in outgrowths at virgin (8 weeks after transplantation), P14 and L1 were analyzed by flow cytometry. Values represent the average ± SD (virgin, *n* = 3; P14, *n* = 4; L1, *n* = 3). **e** Whole-mount analysis of the mammary gland in #2 fat pads of recipient wild-type mice and the outgrowths of transplanted TRAF6-He and TRAF6-KO epithelia in cleared fat pads at P14 and L1. Scale bars, 500 μm

To investigate the role of TRAF6 in mammary gland development during virgin and later stages, a cleared mammary fat pad transplantation assay^28^ was performed, because TRAF6-KO mice die before virgin stage (at 2–3 weeks old)^26^. Fat pads close to the #4 nipple of 7- to 14-day-old female *Traf6*^+/–^ (TRAF6-He) and TRAF6-KO mice (donor epithelia) were transplanted into the right and left #4 cleared (gland-free) fat pads of 3- to 4-week-old recipient WT BALB/c mice. The whole-mount analysis of the fat pads 8 weeks after transplantation in virgin mice revealed that the entire fat pads donated from TRAF6-He and TRAF6-KO mice were similarly filled with primary and secondary ducts with side branches (Fig. 2a and Supplementary Fig. 2). The mammary glands of the recipient WT mice were morphologically indistinguishable from those derived from the transplanted fat pads (Fig. 2a and Supplementary Fig. 2). Moreover, hematoxylin and eosin (H&E) staining (Fig. 2b, Virgin) and immunohistochemical staining for Keratin 5 (Krt5), a basal cell marker, and E-cadherin (E-cad), a luminal cell marker^29^ (Fig. 2c, Virgin), revealed normal organization of the two epithelial layers in the absence of TRAF6. Flow cytometric analyses indicated that the differentiation of mammary epithelia was normal in transplanted TRAF6-KO mammary glands (Fig. 2d, Virgin). These results indicate that TRAF6 is dispensable for mammary gland development in virgin stage.

### TRAF6 is essential for pregnancy-induced alveologenesis

To investigate the role of TRAF6 in mammary gland development during pregnancy, recipient mice were bred 5 weeks after transplantation and the transplanted fat pads were subsequently analyzed on pregnancy day 14 (P14) and lactation day 1 (L1). The whole-mount analysis revealed the pregnancy-induced proliferation of mammary epithelia and sprouting of alveolar buds in both transplanted TRAF6-He and recipient WT mammary glands of P14 mice (Fig. 2e). Further epithelium proliferation and terminal differentiation of the alveolar buds resulted in fully developed lobuloalveolar structures, which blanketed most of the dilated ducts at L1 in the presence of TRAF6 (Fig. 2e). Whereas alveolar structures were formed at P14 and L1 in transplanted TRAF6-KO glands, the magnitude of these changes was less prominent than that in transplanted TRAF6-He mammary glands (Fig. 2e), which was similarly observed in RANKL- or RANK-deficient mice^9^. A histological analysis also revealed that the TRAF6 deficiency resulted in slightly fewer alveolar structures at P14 and severely impaired lobuloalveolar development at L1 (Fig. 2b, P14 and L1). However, the TRAF6 deficiency had no obvious effect on ductal organization (Fig. 2c, P14 and L1) or the basal and luminal cell population sizes (Fig. 2d, P14 and L1). The density of side branching, unchanged between virgin and P14, was not affected by TRAF6 deficiency (Supplementary Fig. 2). Taken together, these results indicate that TRAF6 is required for the expansion of both types of epithelial cells, leading to alveolar development during pregnancy.

### TRAF6 maintains MaSCs and LPCs during the virgin stage

**Fig. 3:**
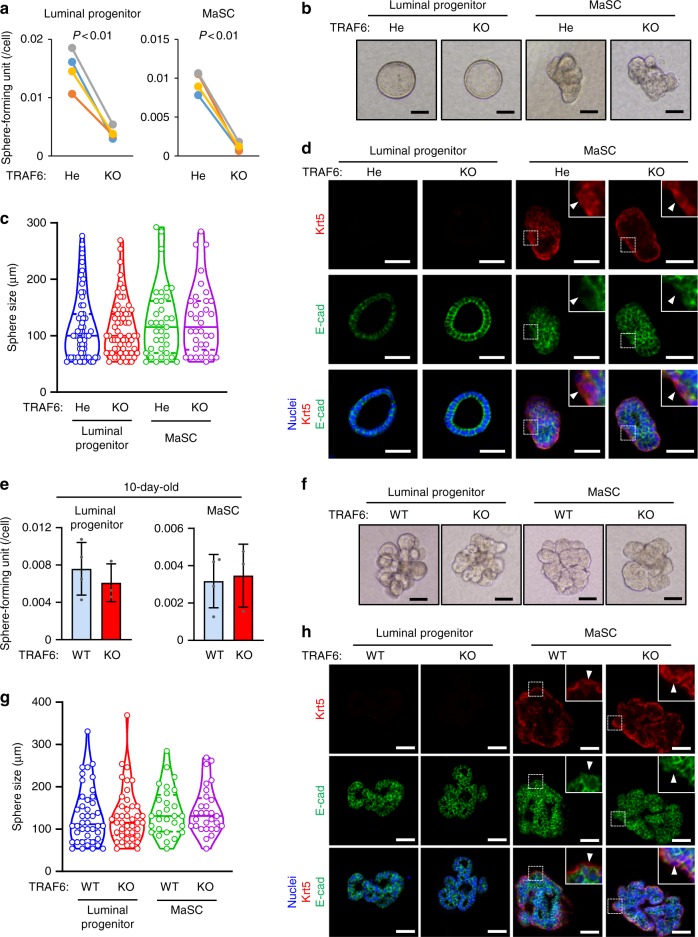
TRAF6 is required for maintaining mammary stem cells (MaSCs) and luminal progenitor cells (LPCs). **a** In vitro limiting-dilution assay of luminal and basal cells isolated from outgrowths of transplanted TRAF6-He and TRAF6-KO epithelia in recipient mice in the virgin stage. Isolated cells were cultured in ultra-low adherent plates at densities of 1000 to 31 cells/well. At 16 days after seeding, spheres (>50 μm) were counted to calculate the sphere-forming unit (TRAF6-He and TRAF6-KO, *n* = 4/group). **b** Morphology of the spheres in **a**. **c** Size distribution of the spheres in **a**. **d** Immunostaining of the spheres in **a** with antibodies specific to Keratin 5 (Krt5, red) and E-cadherin (E-cad, green). Nuclei were stained with Hoechst 33342 (blue). Insets show higher magnification views of the area indicated by dotted boxes. Arrowheads indicate Krt5^high^E-cad^low^ basal cells. Scale bars, 50 μm. **e** In vitro limiting-dilution assay of luminal and basal cells isolated from mammary tissue of 10-day-old (prepubertal) TRAF6-WT and TRAF6-KO female mice. Isolated cells were cultured in ultra-low adherent plates at densities of 1000–30 cells/well for luminal cells and 700–20 cells/well for basal cells. At 16 days after seeding, spheres (>50 μm) were counted to calculate the sphere-forming unit (TRAF6-WT, *n* = 4; TRAF6-KO, *n* = 3). **f** Morphology of the spheres in **e**. **g** Size distribution of the spheres in **e**. **h** Immunostaining of the spheres in **e** with antibodies sp**e**cific to Keratin 5 (Krt5, red) and E-cadherin (E-cad, green). Nuclei were stained with Hoechst 33342 (blue). Insets show higher magnification views of the area indicated by dotted boxes. Arrowheads indicate Krt5^high^E-cad^low^ basal cells. Scale bars, 50 μm

MaSCs are crucial for pregnancy-induced mammary gland development^30,31^. In addition, luminal progenitor cells (LPCs), which are generated from MaSCs, differentiate into luminal cells, which in turn, enhance MaSC proliferation by expressing RANKL^10^. Therefore, we next investigated whether TRAF6 deficiency affected MaSC and LPC populations. MaSCs and LPCs can grow clonally and form lineage-specific mammospheres in 3D Matrigel in vitro cultures^32^. Therefore, luminal and basal cell populations, which contained LPCs and MaSCs, respectively, were sorted from outgrowths developed from transplanted TRAF6-He and TRAF6-KO fat pads in the virgin stage (Supplementary Fig. 3) and subjected to limiting-dilution assays on Matrigel to determine colony-forming frequency (Supplementary Fig. 4A). The frequency of luminal and basal cell-derived colonies was significantly reduced in the absence of TRAF6 (Fig. 3a). However, their morphology and size distributions were not affected in the absence of TRAF6 (Fig. 3b, c). A lineage marker analysis of LPC- and MaSC-derived colonies revealed that spatial expression patterns of Krt5 and E-cad were indistinguishable in the presence or absence of TRAF6 (Fig. 3d). Furthermore, Krt5^low^E-cad^high^ luminal cells were surrounded by Krt5^high^E-cad^low^ basal cells in MaSC-derived colonies, indicating that MaSCs are bipotent irrespective of TRAF6 expression (Fig. 3d, MaSC panels). Interestingly, lack of TRAF6 did not affect the number of LPC and MaSC colonies derived from epithelial cells of prepubertal glands (Fig. 3e–h and Supplementary Fig. 4B), indicating that TRAF6 is dispensable for the generation of MaSCs and LPCs in the prepubertal stage. Thus, TRAF6 is likely to be crucial for the in vivo maintenance rather than the generation of MaSCs and LPCs.

### TRAF6 is not essential for luminal cell maturation

**Fig. 4:**
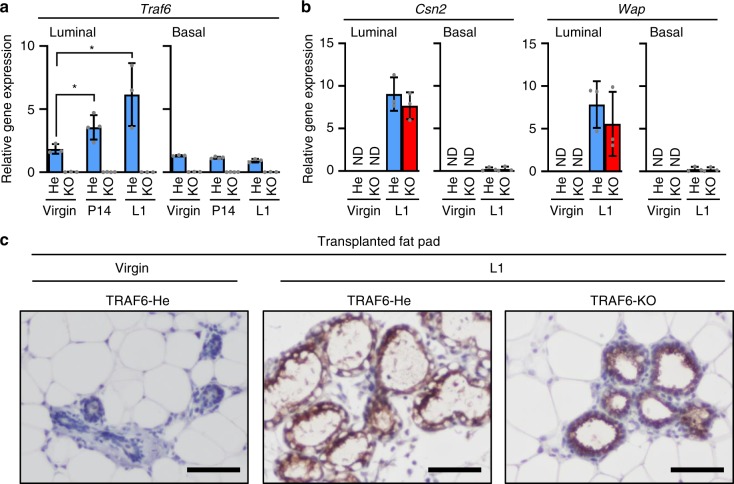
TRAF6 is not essential for pregnancy-induced luminal cell maturation and milk production. **a** Real-time RT-qPCR analysis of *Traf6* expression in luminal and basal cells isolated from outgrowths developed from TRAF6-He and TRAF6-KO epithelia in recipient mice in virgin stage, P14 and L1. Values are means ± SD (Virgin, *n* = 3; P14, *n* = 4; L1, *n* = 3; *p < 0.05). **b** Real-time RT-qPCR analysis of *Csn2* and *Wap* expression in luminal and basal cells isolated from outgrowths developed from TRAF6-He and TRAF6-KO epithelia in recipient in virgin L1. Values are means ± SD (Virgin and L1, *n* = 3/group). **c** Immunostaining of outgrowths in cleared fat pads of recipient mice in virgin and L1 stages using milk-specific antibody. Nuclei were stained with hematoxylin. Scale bars, 200 μm

The expression of β-casein, a major milk protein, is impaired in the absence of RANKL or RANK, indicating that RANK signaling is required for both lobuloalveolar development and luminal cell maturation^9^. To elucidate the role of TRAF6 in luminal cell maturation, the expression of milk-related genes in luminal and basal cells sorted from virgin, P14, and L1 transplanted fat pads was analyzed. The expression of *Traf6* was significantly induced during pregnancy in luminal epithelial but not basal cells, suggesting the critical role of TRAF6 in luminal cell function (Fig. 4a). Nevertheless, the expression per luminal cell of milk-related genes, including casein beta (*Csn2*) and whey acidic protein (*Wap*) (Fig. 4b), and that of milk protein (Fig. 4c) were normally induced in the absence of TRAF6, indicating that TRAF6 is not essential for pregnancy-induced luminal cell maturation. As TRAF6-KO glands showed impaired alveologenesis (Fig. 2b–e), they contained a smaller number of luminal cells compared with TRAF6-WT glands. Therefore, milk production per gland is likely to be significantly reduced in TRAF6-KO glands, leading to insufficient lactation.

### TRAF6 promotes epithelial cell growth during pregnancy

**Fig. 5:**
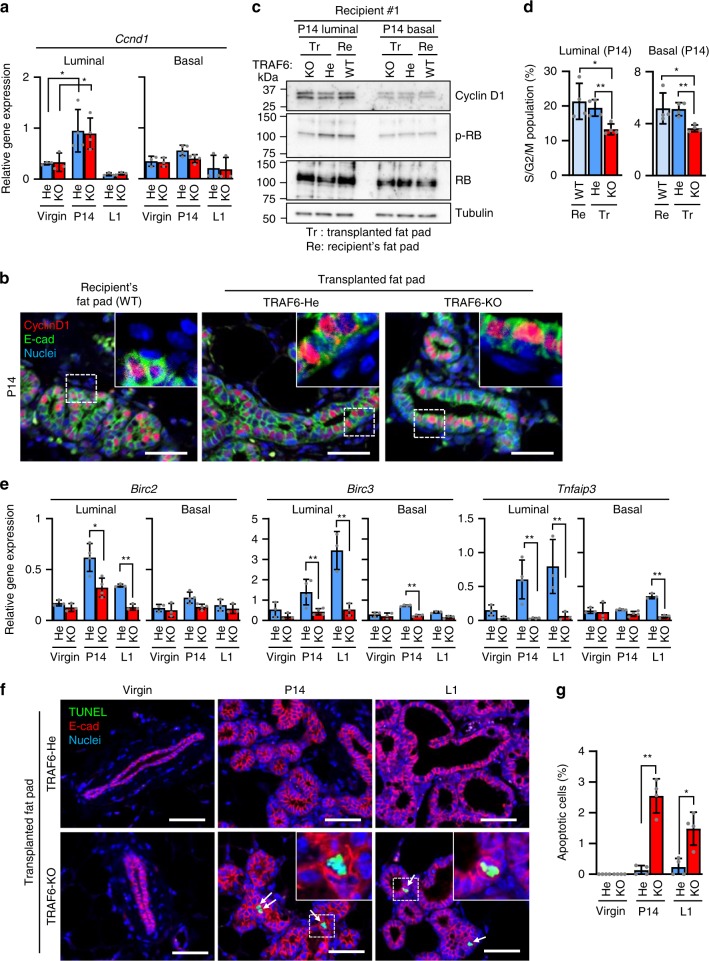
TRAF6 promotes G1/S transition and cell survival during pregnancy. **a** Real-time RT-qPCR analysis of Cyclin D1 mRNA (*Ccnd1*) expression in luminal and basal cells isolated from outgrowths developed from TRAF6-He and TRAF6-KO epithelia in recipient mice. Values are means ± SD (Virgin, *n* = 3; P14, *n* = 4; L1, *n* = 3; *p < 0.05). **b** Immunostaining of the mammary gland in #2 fat pads of recipient wild-type mice and the outgrowths derived from transplanted TRAF6-He and TRAF6-KO epithelia in cleared fat pads at P14 with antibody specific to Cyclin D1 (red) and E-cadherin (green). Nuclei were stained with Hoechst 33342 (blue). Insets show higher magnification views of the area indicated by dotted boxes. Scale bars, 100 μm. **c** Western blotting analysis of Cyclin D1, p-RB, and RB in luminal and basal cells isolated from outgrowths in recipient WT mice (#1) at P14. Tr: Transplanted fat pad; Re: Recipient’s fat pad. **d** S/G2/M phase population was analyzed in luminal and basal cells isolated from outgrowths in recipient mice at P14. Values are means ± SD (*n* = 4; *p < 0.05 and **p < 0.01). **e** Real-time RT-qPCR analysis of *Birc2*, *Birc3*, and *Tnfaip3* expression in luminal and basal cells isolated from outgrowths developed from TRAF6-He and TRAF6-KO epithelia in recipient mice. Values are means ± SD (Virgin, *n* = 3; P14, *n* = 4; L1, *n* = 3; *p < 0.05 and **p < 0.01). **f** TUNEL staining of outgrowths in cleared fat pads of recipient mice. Tissues were stained with anti-E-cadherin antibody (E-cad, red) after TUNEL staining. Arrows indicate TUNEL-positive cells (green). Nuclei were stained with Hoechst 33342 (blue). Insets show higher magnification views of the area indicated by dotted boxes. Scale bars, 100 μm. **g** Population of TUNEL-positive epithelial cells in **f**. Values are means ± SD (virgin, *n* = 3; P14, *n* = 3; L1, *n* = 3; *p < 0.05 and **p < 0.01)

RANKL-induced Cyclin D1 expression is crucial for pregnancy-induced mammary epithelial cell proliferation^18^. As TRAF6 is downstream of RANK^25^ and TRAF6 deficiency phenocopies RANK and IKKα deficiencies in lobuloalveolar expansion during pregnancy^9,18^, Cyclin D1 induction was analyzed in the absence of TRAF6. Unexpectedly, Cyclin D1 mRNA (*Ccnd1*) was normally upregulated in TRAF6-deficient luminal cells at P14, but its upregulation was not observed in basal cells even in the presence of TRAF6 (Fig. 5a). Consistently, lack of TRAF6 did not significantly affect Cyclin D1 protein expression at P14 in both luminal and basal cells, whereas its expression levels were higher in E-cad-positive luminal cells (Fig. 5b, c and Supplementary Fig. 5). Interestingly, TRAF6-dependent enhancement of retinoblastoma (RB) phosphorylation was observed only in luminal cells (Fig. 5c), even though lack of TRAF6 significantly reduced the S/G2/M population in both luminal and basal epithelia at P14 (Fig. 5d). These data lead to two important suggestions. First, the mechanisms underlying the TRAF6-dependent proliferation likely differ between luminal and basal cells, which may be related to the luminal-specific induction of TRAF6 (Fig. 4a) and RANKL (Supplementary Fig. 6). Second, TRAF6 is involved in the G1 to S-phase transition of luminal cells by enhancing RB phosphorylation through a mechanism distinct from Cyclin D1 induction, whereby the RANK-IKKα pathway enhanced RB phosphorylation. Expression of other cyclins and cyclin-dependent kinases that promote the G1 to S-phase transition, including *Ccnd2*, *Ccne1*, *Ccne2, Cdk2*, *Cdk4*, and *Cdk6*, was not reduced in the absence of TRAF6, whereas, among cdk inhibitors, *Ink4c* and *Arf* mRNAs were upregulated in TRAF6-KO luminal cells at P14 (Supplementary Fig. 7). As *Ink4c* and *Arf* are reported as growth suppressor genes in mammary gland epithelial cells during pregnancy^33,34^, these CDK inhibitors might be involved in the reduction of RB phosphorylation by TRAF6 deficiency.

### TRAF6 promotes cell survival during pregnancy

As the anti-apoptotic role of TRAF6 has been reported^35,36^, we next assessed whether TRAF6 plays any roles in cell survival in mammary gland development. Reverse transcriptase-qPCR (RT-qPCR) analyses of various anti-apoptotic genes revealed that the expression levels of cIAP1 (*Birc2*), cIAP2 (*Birc3*), and A20 (*Tnfaip3*) (Fig. 5e), but not of XIAP (*Birc4*) and Survivin (*Birc5*) (Supplementary Fig. 8A), were significantly upregulated during pregnancy in TRAF6-He luminal cells. However, this upregulation was significantly suppressed at P14 and L1 in the absence of TRAF6 (Fig. 5e). Consistently, a significant increase in terminal deoxynucleotidyl transferase dUTP nick end labeling (TUNEL)-positive cells in the E-cad-positive luminal lineage was observed in TRAF6-KO mammary glands at P14 and L1 (Fig. 5f, g). Interestingly, the expression of two other anti-apoptotic genes, Bcl-2 (*Bcl2*) and Bcl-X_L_ (*Bcl2l1*), which are regulated by the inhibitor of DNA binding 2 (Id2) pathway downstream of RANK^37,38^, was not affected by TRAF6 deficiency (Supplementary Fig. 8B). Considering that Id2 is an essential factor for the pregnancy-induced expansion of mammary epithelial cells^37,38^, Id2 and TRAF6 could cooperatively regulate epithelial cell survival during pregnancy by inducing the expression of different sets of anti-apoptotic genes.

### TRAF6 activates canonical NF-κB and AKT during pregnancy

**Fig. 6:**
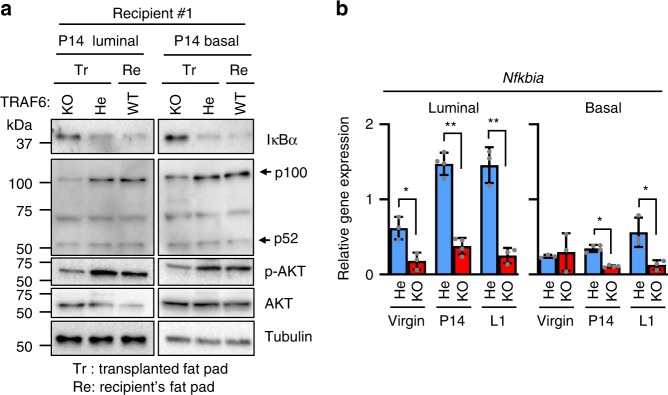
TRAF6 selectively activates canonical NF-κB and AKT pathways in mammary epithelial cells during pregnancy. **a** Western blotting analysis of IκBα, p100, p52, phosphorylated AKT (p-AKT), and AKT expression in luminal and basal cells isolated from the mammary gland in #2 fat pads of recipient WT mice (#1) and the outgrowths derived from transplanted TRAF6-He and TRAF6-KO epithelia in cleared fat pads at P14. Tr: Transplanted fat pad; Re: Recipient’s fat pad. **b** Real-time RT-qPCR analysis of *Nfkbia* (IκBα mRNA) expression in luminal and basal cells isolated from outgrowths in recipient mice. Values are means ± SD (Virgin, *n* = 3; P14, *n* = 4; L1, *n* = 3; *p < 0.05 and **p < 0.01)

RANK activates two distinct NF-κB pathways, the canonical and noncanonical pathways^11,12^. However, the precise distribution of the roles of these two NF-κB pathways in RANKL-induced mammary epithelial cell proliferation had not been elucidated. Therefore, the involvement of TRAF6 in the two pathways needed to be addressed. As RANKL was exclusively expressed in luminal cells and RANK was expressed in both luminal and basal cells irrespective of TRAF6 expression (Supplementary Fig. 6), both luminal and basal cells could be stimulated by RANKL during pregnancy in the absence or presence of TRAF6. Thus, cell lysates and total RNA extracts were prepared from sorted luminal and basal cells of transplanted mammary glands at P14 and analyzed for the levels of IκBα protein and IκBα mRNA (*Nfkbia*) expression as canonical pathway indicators and processing of p100 to p52 as a noncanonical pathway indicator^13^. The amount of IκBα protein was significantly higher in TRAF6-KO than in TRAF6-He luminal cells (Fig. 6a and Supplementary Fig. 9), whereas *Nfkbia* expression was significantly reduced in the absence of TRAF6 (Fig. 6b). Considering that canonical pathway activation induced *Nfkbia* expression and the degradation of IκBα protein, these results strongly suggest that the TRAF6 deficiency abrogated the canonical pathway in luminal cells. As p100 is induced by the canonical pathway, its levels in TRAF6-KO cells were lower than those in TRAF6-He cells (Fig. 6a and Supplementary Fig. 9). Nevertheless, the amounts of processed p52 were similar irrespective of TRAF6 expression (Fig. 6a and Supplementary Fig. 9). Because the noncanonical pathway-mediated gene expression was regulated by the amount of p52, TRAF6 deficiency may not significantly affect the noncanonical pathway-mediated gene expression in luminal cells during pregnancy in vivo. Similar results were observed in basal cells.

Because RANK activates AKT in mammary epithelia^9^, TRAF6-dependent AKT activation was analyzed. The AKT phosphorylation was significantly reduced in the absence of TRAF6 (Fig. 6a and Supplementary Fig. 9). These results indicated that TRAF6 activated AKT pathway in addition to the canonical NF-κB pathway in mammary epithelial cells during pregnancy.

### Canonical and noncanonical pathways cooperate in cell growth

**Fig. 7:**
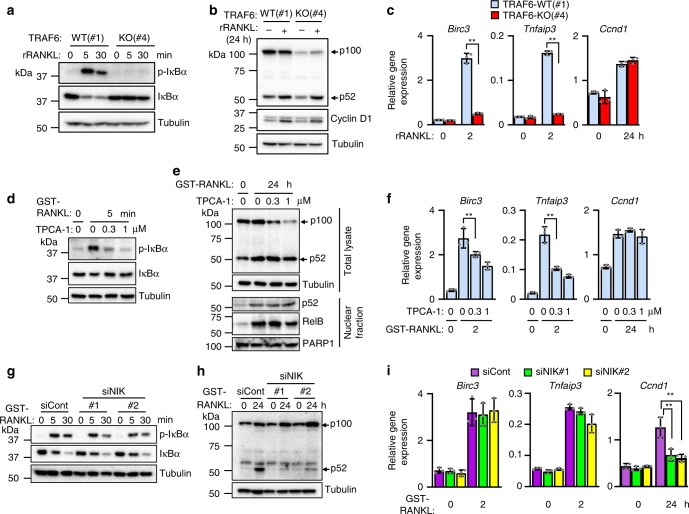
RANK-induced canonical and noncanonical NF-κB pathways have distinct roles in the pregnancy-dependent expansion of mammary epithelial cells. **a** Western blotting analysis of p-IκBα and IκBα. NMuMG-RANK clone#1 (WT) and #4 (KO) were pretreated with serum-reduced DMEM (1% FBS) for 8 h and then stimulated with rRANKL. **b** Western blotting analysis of p100, p52, and Cyclin D1 expression. NMuMG-RANK clones were pretreated as in **a** and then stimulated with rRANKL for 24 h. **c** Real-time RT-qPCR analysis of *Birc3*, *Tnfaip3*, and *Ccnd1* expression in NMuMG-RANK clones. Total RNA was prepared and subjected to RT-qPCR. Values are means ± SD (*n* = 3; **p < 0.01). **d**, **e** Effect of TPCA-1, an IKKβ inhibitor, on canonical (**d**) and noncanonical (**e**) pathways. NMuMG-RANK parent cells were pretreated as in **a** and then treated with TPCA-1 alone for 30 min followed by stimulation with GST-RANKL with TPCA-1. Whole-cell lysates were prepared and subjected to western blotting analysis. **f** Real-time RT-qPCR analysis of *Birc3*, *Tnfaip3*, and *Ccnd1*. NMuMG-RANK parent cells were pretreated as in **a** and then treated with TPCA-1 alone for 30 min followed by GST-RANKL stimulation with TPCA-1. Total RNA was prepared and subjected to RT-qPCR. Values are mean ± SD (*n* = 3; **p < 0.01). **g**, **h** Effect of NIK-knockdown on canonical (**g**) and noncanonical (**h**) pathways. NMuMG-RANK parent cells were transfected with control siRNA or two distinct siRNAs against *Map3k14* (NIK, #1 and #2) for 24 h. Cells were then pretreated as in **a** followed by GST-RANKL stimulation. Whole-cell lysates were prepared and subjected to western blotting analysis. **i** Real-time RT-qPCR analysis of *Birc3*, *Tnfaip3*, and *Ccnd1* expression. Control or NIK siRNA-treated NMuMG-RANK cells were pretreated as in **a** and then stimulated with GST-RANKL. Total RNA was prepared and subjected to RT-qPCR. Values are means ± SD (*n* = 3, **p < 0.01)

To precisely clarify the roles of the canonical and noncanonical NF-κB pathways in the pregnancy-dependent expansion of mammary epithelial cells, NMuMG mouse mammary epithelial cells^39^ lacking TRAF6 were generated using CRISPR/Cas9-mediated genome engineering^40^. Three independent clones of each genotype (TRAF6-WT (#1, #2, #3) and TRAF6-KO (#4, #5, #6)) were established (Supplementary Fig. 10A) and mouse RANK cDNA was then introduced into each clone and parent NMuMG cells to generate NMuMG-RANK cells (Supplementary Fig. 10B). TRAF6 KO abrogated the RANKL-induced IκBα phosphorylation (Fig. 7a and Supplementary Fig. 10C), but scarcely affected the amount of processed p52 (Fig. 7b and Supplementary Fig. 10D). Furthermore, TRAF6 KO suppressed *Birc3* and *Tnfaip3* induction but not that of *Ccnd1* (Fig. 7c and Supplementary Fig. 10E). These data indicate that the effects of TRAF6 deficiency on NF-κB signaling and its target gene expression in mammary epithelia during pregnancy (Fig. 5a–e and 6a) were reproduced in NMuMG-RANK cells. Therefore, the NMuMG-RANK cell line is a suitable model for investigating the molecular mechanisms of RANKL-induced mammary epithelial cell expansion and survival in vivo. To further confirm the role of the canonical pathway, parent NMuMG-RANK cells were treated with TPCA-1, a selective inhibitor of IKKβ^41^. Treatment with 0.3 and 1 μM TPCA-1 significantly inhibited RANKL-induced IκBα phosphorylation (Fig. 7d) without significantly affecting the amounts of processed p52 or the nuclear translocation of p52/RelB, even though the amount of p100 was reduced (Fig. 7e). Furthermore, TPCA-1 treatment significantly reduced *Birc3* and *Tnfaip3* expression, but *Ccnd1* induction was unaffected (Fig. 7f). This observation indicates that TPCA-1 selectively inhibited the RANKL-induced canonical pathway but did not affect the noncanonical pathway-mediated gene expression. In contrast, small interfering RNA (siRNA)-mediated silencing of NIK, an essential kinase in the noncanonical but not the canonical pathway^13^ in parent NMuMG-RANK cells, did not affect the normal RANKL-induced IκBα phosphorylation, whereas p52 generation was significantly inhibited (Fig. 7g, h). Under this conditions, RANKL-induced *Ccnd1* expression was severely blocked, whereas anti-apoptotic genes were normally upregulated (Fig. 7i). These data clearly indicate that the anti-apoptotic genes *Birc3* and *Tnfaip3* are induced by a TRAF6-mediated canonical pathway, whereas *Ccnd1* is induced by the NIK-mediated noncanonical pathway and the induction is not affected by TRAF6 deficiency.

### The RANK-TRAF6-AKT-RB axis promotes cell growth

**Fig. 8:**
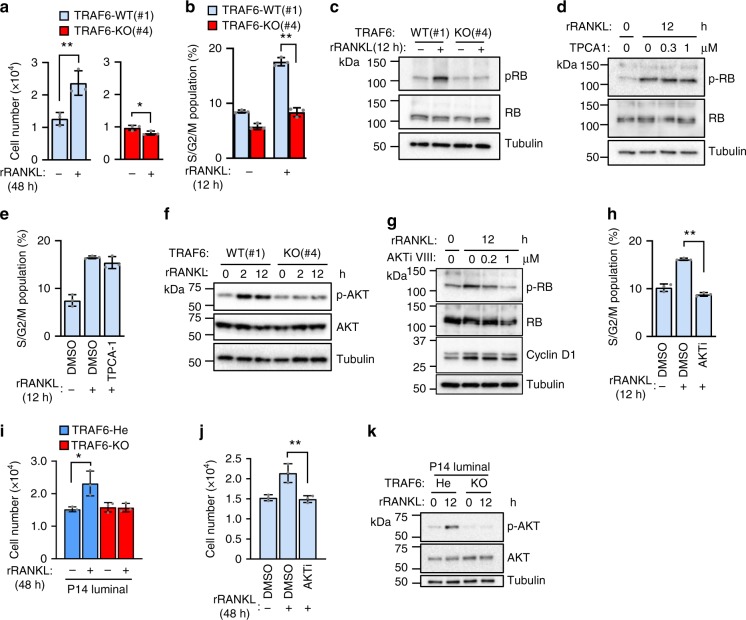
RANKL-induced epithelial cell proliferation is mediated by TRAF6-dependent AKT activation leading to RB phosphorylation. **a** RANK-TRAF6 pathway-dependent proliferation of NMuMG-RANK clone #1(WT) and #4(KO). Cells were pretreated with serum-reduced DMEM (1% FBS) for 8 h and then stimulated with RANKL for 48 h. **b** S/G2/M phase population. Cells were pretreated as in **a** and then stimulated with RANKL for 12 h. **c** Western blotting analysis of RB phosphorylation. Cells were treated as in **b**. **d** Western blotting analysis of RB phosphorylation. NMuMG-RANK parent cells were pretreated as in **a** and then treated with TPCA-1 alone for 20 min followed by treatment with RANKL together with TPCA-1. **e** S/G2/M phase population of NMuMG-RANK parent cells treated as in **d**. **f** Western blotting analysis of AKT phosphorylation. NMuMG-RANK clones were pretreated as in **a** and then stimulated with RANKL. **g** Western blotting analysis of RB phosphorylation in AKT inhibitor-treated NMuMG-RANK parent cells. Cells were pretreated as in **a** and then treated with AKT inhibitor VIII (AKTi VIII) for 60 min followed by treatment with RANKL together with AKTi VIII. **h** S/G2/M phase population of the AKTi VIII-treated NMuMG-RANK parent cells. Cells were treated as in **g**. **i** RANK-TRAF6-AKT pathway-dependent proliferation of primary luminal epithelial cells. Primary luminal cells were isolated from outgrowths of transplanted TRAF6-He and TRAF6-KO epithelia in recipient mice of P14. Cells were treated with or without RANKL for 48 h. **j** Effect of AKT inhibition on primary luminal epithelial cells proliferation. Primary luminal cells isolated from mammary glands of TRAF6-WT P14 mice were RANKL stimulated for 48 h with or without AKTi VIII. **k** Western blotting analysis of AKT phosphorylation in TRAF6-He or TRAF6-KO primary luminal epithelial cells after RANKL stimulation. Cells were isolated as in **i**. Values are means ± SD (*n* = 3, *p < 0.05 and **p < 0.01) (**a**, **b**, **e**, **h**, **i**, **j**)

To further clarify the TRAF6-dependent pathway in cell proliferation, the RANKL-dependent proliferation of NMuMG-RANK cells was analyzed. The enhancement of cell proliferation was clearly TRAF6 dependent (Fig. 8a, b and Supplementary Fig. 11A, B). Consistently, RANKL-dependent enhancement of RB phosphorylation (Fig. 8c and Supplementary Fig. 11C) was TRAF6 dependent. These results strongly suggest that NMuMG-RANK cells maintain the RANK-TRAF6-RB-axis-induced cell growth mechanisms similar to those for the pregnant-dependent proliferation of mammary epithelial cells in vivo. Although TRAF6 is required for the RANKL-dependent enhancement of cell proliferation, treatment with TPCA-1, a selective inhibitor of the canonical pathway^41^, did not block the RANKL-induced enhancement of RB phosphorylation (Fig. 8d) and expansion of S/G2/M population (Fig. 8e). Therefore, we focused on AKT, because AKT activation was TRAF6-dependent in transplanted mammary epithelia during pregnancy (Fig. 6a). Similarly, RANKL-induced AKT activation was TRAF6 dependent in NMuMG-RANK cells (Fig. 8f and Supplementary Fig. 11D). Furthermore, inhibition of AKT activation by the AKT inhibitor VIII (AKTi VIII)^42,43^ (Supplementary Fig. 11E) completely suppressed the RANKL-induced enhancement of RB phosphorylation (Fig. 8g) and expansion of the S/G2/M population (Fig. 8h). To further confirm the critical role of the RANK-TRAF6-AKT pathway in luminal cell proliferation, primary luminal cells purified from P14 TRAF6-He and TRAF6-KO transplanted fat pads were stimulated with RANKL. The RANKL-induced primary luminal cell proliferation required both TRAF6 and TRAF6-dependent AKT activation (Fig. 8i–k). These data clearly indicate that the TRAF6-AKT pathway is crucial for RANKL-induced cell proliferation, although canonical NF-κB activation is dispensable for proliferation.

## Discussion

**Fig. 9:**
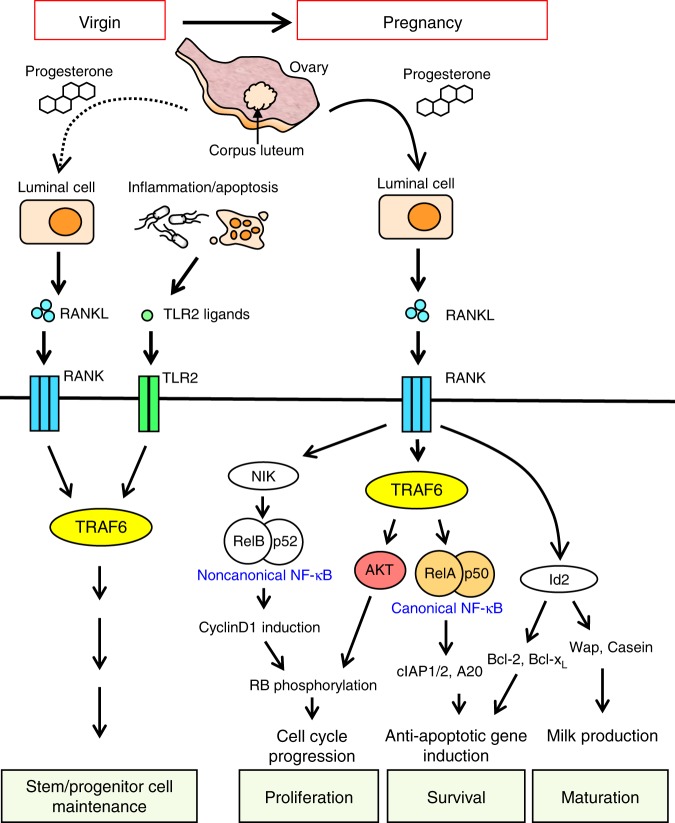
Model illustrating TRAF6-mediated mammary gland development during the virgin stage and pregnancy. See text for details

As the most important function of the mammary gland is to produce milk to nourish suckling offspring and protect them from diseases^1^, both the quality and quantity of mammary epithelial cells are important. Cell differentiation and maturation have a large role in quality control, and cell proliferation and survival contribute greatly to maintaining their quantity. During virgin and pregnancy stages, progesterone, a hormone released from the corpus luteum in the ovary, activates the progesterone receptor expressed in luminal cells to induce RANKL expression. The expressed RANKL then stimulates RANK on both luminal and basal cells to control the quality and quantity of mammary epithelial cells^9^. RANK activates several signaling pathways including the canonical and noncanonical NF-κB, PI3K/AKT, and Id2 pathways (Fig. 9). Although the Id2 activation is known to promote cell survival via *Bcl-2* and *Bcl-xL* expression and cell maturation to induce milk genes^37,38^, the distribution of the roles played by the canonical and noncanonical pathways and the PI3K/AKT pathway remains unclear. In this study, we distinguished the roles of these pathways in mammary gland development by analyzing transplanted TRAF6-KO mammary glands and cultured mammary epithelial NMuMG cells. These experiments provide evidence to support a model in which the two distinct modes of cooperation between the TRAF6-dependent and -independent pathways harmonize to promote pregnancy-dependent expansion of mammary epithelia (Fig. 9). First, the TRAF6-dependent canonical NF-κB pathway induces the expression of *Birc2/3* and *Tnfaip3* to block cell death in cooperation with the TRAF6-independent Id2 pathway-mediated induction of *Bcl-2* and *Bcl-xL* expression. Second, *Ccnd1* induction mediated by the TRAF6-independent noncanonical pathway promotes RB phosphorylation in concert with the TRAF6-dependent PI3K/AKT activation, which leads to cell proliferation.

Our data from the precise analyses of sorted luminal and basal cell populations strongly suggest distinct proliferative mechanisms of basal compared with luminal cells, although TRAF6 is required for pregnancy-induced proliferation of both cell types. Although RANK-induced canonical NF-κB and AKT activation were clearly TRAF6 dependent in both basal and luminal cells, TRAF6-dependent induction of anti-apoptotic genes and increase in p-RB levels during pregnancy were not observed in basal cells. Furthermore, RANKL expression and the significant induction of Cyclin D1 and TRAF6 were only observed in luminal cells. These discrepancies between luminal and basal cells strongly suggest that molecular mechanisms of the basal cell expansion during pregnancy may not fit our present model shown in Fig. 9. Consistent with this notion, NMuMG cells, which provided critical data supporting our model, are classified as luminal cells. Based on these results, one could speculate that strong induction of TRAF6 in luminal cells is required for RB phosphorylation and anti-apoptotic gene induction to increase the number of luminal cells, whereas TRAF6 in basal cell does not play a major role in the proliferation and survival of basal cells. As RANKL and Wnt4 are exclusively expressed in luminal cells and stimulate basal cells to proliferate^10,44,45^, increasing luminal cell numbers, which is cell-autonomously mediated by TRAF6, could result in enhanced expression of RANKL and Wnt4, thereby promoting further basal cell proliferation. In other words, reduced number of luminal cells resulting from TRAF6 deficiency lead to reduced expression of RANKL and Wnt4, and consequently limited proliferation of basal cells.

In addition to its role in epithelial cell proliferation, TRAF6 is crucial for the maintenance of MaSCs and LPCs during the virgin stage but dispensable for their generation in the prepubertal stage. As these cells have proliferative potential during pregnancy^30,31^, this role of TRAF6 might partly explain the significant reduction in epithelial cell numbers during pregnancy in TRAF6-KO glands compared with TRAF6-He glands. The RANK and TLR2 signaling pathways, which both use TRAF6 to transduce signals^11^, are involved in maintaining MaSC and LPC populations during the virgin stage^10,46^ (Fig. 9). Thus, TRAF6 plays a cell-autonomous role in MaSC maintenance in basal cells, which might be different from the role TRAF6 plays in basal cell proliferation.

RANK signaling inactivation by the anti-RANKL antibody reduced the number of MaSCs by ~50% but had no effect on the LPC population^10^. In contrast, TLR2 deficiency slightly decreased the LPC population but markedly reduced the number of MaSCs^46^. Therefore, the significant decrease in the number of MaSCs induced by TRAF6 deficiency could be explained by the concomitant inactivation of RANK and TLR2 pathways. However, the significant reduction in LPCs observed in TRAF6-KO glands suggest that the two pathways might be largely redundant or other TRAF6-mediated signaling pathways could be involved in LPC maintenance. Integrin β1 (CD29) deficiency in mammary epithelia did not affect glandular development in virgins but reduced the MaSC population and impaired gland development during pregnancy^30^, which is phenotypically similar to the TRAF6-KO glands. However, integrin β1 expression in epithelial cells in virgin stage and at P14 was not affected in TRAF6-KO glands (Supplementary Fig. 12). Further studies are required to elucidate the mechanism underlying TRAF6-mediated regulation of MaSC and LPC populations.

NF-κB is also crucial for breast cancer development by enhancing cell survival, proliferation, and invasion, and maintaining cancer stem cell populations^47–52^. It has been reported that hormone-induced expression of *Birc3* and *Tnfaip3*, target genes of the RANK-TRAF6-mediated canonical NF-κB pathway in normal epithelial cells, in luminal-like breast cancers are involved in cancer cell survival and chemoresistance^53,54^. Moreover, aberrant activation of AKT, which is activated by the RANK-TRAF6 pathway in the proliferation of normal epithelial cells, is important for breast cancer development by promoting cell proliferation^55^. These results led us to hypothesize that the RANK-TRAF6 axis is likely involved in RANK-dependent breast cancer malignancy^56–59^. Consistent with this hypothesis, previous studies have suggested that TLR2 and RANK-mediated MaSC and LPC maintenance are important for WNT- and carcinogen-induced tumorigenesis, respectively^46,60^.

Further experiments would be required to elucidate the precise roles of TRAF6 in normal mammary gland and breast cancer development. However, our present elucidation of the anti-apoptotic and proliferative role of the TRAF6-mediated canonical NF-κB and AKT pathways downstream of RANK might contribute to the development of therapeutic strategies for malignant breast cancers.

## Methods

### Antibodies, plasmids, and reagents

The antibodies used were as follows: anti-CD24-FITC (#11-0242-85), anti-ScaI-PE (#12-5981-82), and anti-CD140b-biotin (#13-1402-82, eBioscience, San Diego, CA, USA); anti-EpCAM-FITC (#118208), anti-CD61-APC (#104316), and anti-CD29-PE (#102207, BioLegend, San Diego, CA, USA); anti-CD49f-PE/Cy5 (#551129, BD Pharmingen, San Diego, CA, USA); mouse epithelial cell enrichment cocktail (1:20, mixture of biotinylated antibodies against CD45, TER119, BP-1, and CD31; STEMCELL Technologies, Vancouver, Canada); PE-CF594-streptavidin (#562284, BD Biosciences, San Jose, CA, USA); anti-keratin5 (Covance, Denver, PA, USA); anti-E-cad (#610181, BD Transduction Laboratories, Lexington, KY, USA); anti-milk-specific protein, mouse (RAM/MSP, Nordic-MUbio, Susteren, Netherlands); anti-Cyclin D1 (ab134175, Abcam, Cambridge, MA, USA); anti-IκBα (#9242S), anti-p-IκBα (#9246L), anti-p100/p52 (#4882S), anti-RelB (#4922S), anti-RelA (#4764S), anti-HDAC2 (#2540S), anti-p-RB (#8516), anti-p-ATK (#4060), anti-ATK (#4691), and Signal Stain Boost IHC detection reagent (#8114P, Cell Signaling Technology, Danvers, MA, USA); anti-Tubulin (CP06, Millipore, Darmstadt, Germany); anti-TRAF6 (sc-7221), and anti-PARP-1 (sc-25780, Santa Cruz Biotechnology, Santa Cruz, CA, USA); and anti-TRAF6 (Ab33915) and anti-RB (Ab181616, Abcam). Mouse cDNA encoding RANK were generated using PCR and inserted into the retroviral vector pMXs obtained from T. Kitamura (University of Tokyo, Japan). For the CRISPR/Cas9-mediated TRAF6 KO, sgRNA sequence 5′-GCT GGA GAG GTT CCC GGT GC-3′ were inserted into the eSpCas9 (1.1, #71814, Addgene, eSpCas9[1.1]-mTraf6). The reagents used were as follows: epidermal growth factor (EGF) and basic fibroblast growth factor (bFGF, BD Pharmingen); 7-amino-actinomycin D (7AAD) (Millipore); collagenase (C5138), hyaluronidase (H4272), and DNase I (DN25), TPCA-1 (T1452, Sigma-Aldrich); AKTi VIII (124018, Merck); dispase (#07913) and an EpiCult-B Mouse medium kit (#05610, STEMCELL Technologies); and growth-factor-reduced Matrigel (#356230, BD Biosciences). GST-fused mouse RANKL (GST-RANKL) was purified from *Escherichia coli* BL21 harboring a pGEX-GST-RANKL plasmid using glutathione sepharose 4B (#17-0756-05, GE Healthcare Biosciences, Piscataway, NJ, USA). Next, endotoxin was removed using Affinity Pak Detoxi-Gel Endotoxin Removing Gel (#20344, Pierce, Rockford, IL, USA) according to the manufacturer’s protocol. For RNA interference (RNAi) experiments, all stealth RNAi oligonucleotides including control RNA (#12935-113) were purchased from Thermo Fisher Scientific. Oligonucleotides used for RNAi experiments are described in Supplementary Table 1.

### Cell culture

The NMuMG cell line^39^, a generous gift from Dr. Kentaro Semba (Waseda University, Tokyo Japan), was cultured in Dulbecco’s modified Eagle’s medium (DMEM; Wako, Tokyo, Japan) supplemented with 10% fetal bovine serum (FBS), 100 U mL^−1^ penicillin, and 100 µg mL^−1^ streptomycin at 37 °C exposed to 5% CO_2_.

### Mice

*Traf6*^−/−^ mice on the BALB/c background were established as previously described^24^. All the mice were maintained under specific pathogen-free conditions and were handled in accordance with the Guidelines for Animal Experiments of the Institute of Medical Science, The University of Tokyo (Tokyo, Japan). The day the vaginal plug was observed was designated as day 0 of pregnancy and the parturition day was designated as L1. Plugged females were caged separately and killed on P14 and L1.

### Transfection

NMuMG cells expressing mouse RANK (NMuMG-RANK) were established by retroviral infection with pMXs-mRANK-puro constructed from pMXs-puro retroviral vector, followed by selection with 1 μg mL^−1^ puromycin. siRNA transfection was achieved using Lipofectamine RNAiMAX (Thermo Fisher Scientific) according to the manufacturer’s protocol. Cells were seeded in 24-well plates (1 × 10^5^ cells per well) with siRNA and Lipofectamine RNAiMAX. At 24 h after first transfection, culture medium was changed to serum-reduced DMEM (1% FBS) with siRNA and Lipofectamine RNAiMAX for the second transfection. At 8 h after the second transfection, cells were stimulated with GST-RANKL (100 ng ml^−1^) or recombinant RANKL (rRANKL, 25 ng mL^−1^, #310-01, Peprotech, NJ, USA) and collected for western blotting and real-time RT-qPCR analyses. For the establishment of TRAF6-deficient NMuMG cells, eSpCas9(1.1)-mTraf6 plasmid expressing Cas9 and gRNA was transfected using TurboFect (Thermo Fisher Scientific, San Jose, CA, USA) according to manufacturer’s protocol. Cell clones were generated by limited dilution at 72 h after transfection and analyzed for TRAF6 expression using western blotting analysis. TRAF6-expressing clones (#1, #2, and #3) and TRAF6-deficient clones (#4, #5, and #6) were selected and then infected with retrovirus expressing murine RANK (pMXs-mRANK-puro) followed by puromycin selection to generate RANK-expressing NMuMG clones (#1-#6).

### Reverse transcriptase-qPCR

Total RNA was isolated from primary mammary epithelial cells using a RNeasy Micro kit (Qiagen, Valencia, CA, USA) and from NMuMG-RANK cells with TRIzol reagent (Thermo Fisher Scientific). cDNA was synthesized from 1 μg total RNA using SuperScript VILO (Thermo Fisher Scientific). Real-time RT-qPCR analysis was performed using a Life Technologies 7300 Fast Real-Time PCR system and FastStart Universal SYBR Green Master mix (Roche, Basel, Switzerland). The level of β-actin expression was used to normalize the data. Primers used for real-time RT-qPCR are described in Supplementary Table 2.

### Western blotting analysis

Whole-cell lysates were separated using SDS-polyacrylamide gel electrophoresis and transferred onto polyvinylidene difluoride membranes (Immobilon P, Millipore). Membranes were then incubated with primary antibodies (1:1000). Immunoreactive proteins were visualized with anti-rabbit or anti-mouse IgG conjugated to horseradish peroxidase (GE Healthcare, Piscataway, NJ, USA), followed by processing with an enhanced chemiluminescence detection system (GE Healthcare). Nuclear fractions were prepared as previously described^50^. Full-length blots are presented in Supplementary Fig. 13.

### Flow cytometry

Mammary fat pads were mechanically dissociated using scissors and scalpel, and then digested for 60 min at 37 °C in DMEM/F12 (Wako) supplemented with 5% FBS, 3 mg mL^−1^ collagenase, and 50 μg mL^−1^ hyaluronidase. The resulting suspension was sequentially resuspended in 0.25% trypsin-ethylenediaminetetraacetic acid for 5 min and then in 5 U mL^−1^ dispase with 0.1 mg mL^−1^ DNase I for 5 min, followed by filtration through a 40 µm mesh. To separate hematopoietic cells, red blood cells, endothelial cells, and fibroblasts, single cells were stained with a mouse epithelial enrichment cocktail (a mixture of biotinylated antibodies against CD45, TER119, BP-1, and CD31) and anti-CD140b-biotin (1:100) for 15 min at 4 °C in antibody staining buffer (HANK’s buffer supplemented with 1% FBS). To analyze mammary epithelial cell population in virgin mammary glands, cells were stained with anti-CD24 fluorescein isothiocyanate (FITC) (1:100), anti-CD49f-PE/Cy5 (1:100), anti-CD61-APC (1:100), anti-ScaI-PE (1:100) antibodies, and streptavidin-PE-CF594 (1:100) for 30 min at 4 °C in antibody staining buffer. To analyze the mammary epithelial cell population in P14 and L1 mammary glands, cells were stained with anti-EpCAM-FITC (1:100), anti-CD49f-PECy5 (1:100) antibodies, and PE-CF594-streptavidin (1:100) for 30 min at 4 °C in antibody staining buffer. Dead cells were stained with 0.5 μg mL^−1^ 7AAD. Next, the cells were analyzed using an Epics XL flow cytometer (Beckman Coulter, Brea, CA, USA), FACSVerse flow cytometer, and a FACSAria flow cytometer (BD Biosciences). For cell cycle analysis by quantification of DNA content, sorted primary or NMuMG cells were incubated with Vybrant DyeCycle Green Stain (Thermo Fisher Scientific), a cell-permeable DNA dye, for 30 min at 37 °C and analyzed using a FACSVerse flow cytometer.

### Cleared mammary fat pad transplantation

Mammary fat pad pieces of ~5 mm^3^ in size were dissected from 7- to 14-day-old female TRAF6-KO or TRAF6-He mice on BALB/c background. These pieces were implanted into the inguinal number 4 fat pads of 3- to 4-week-old female BALB/c females cleared of endogenous epithelium, as previously described^28^. The outgrowths produced were analyzed 8 weeks after transplantation into the virgin mice. The recipient mice were bred for 5 weeks after transplantation and then analyzed at P14 and L1.

### Histology, whole-mount staining, and immunostaining

For histological analysis, mammary fat pads were embedded in paraffin, 5 μm sections were cut, and then H&E stained. Mammary glands were whole-mount stained as previously described^61^. For immunostaining, paraffin-embedded sections were dehydrated and antigenic epitopes were exposed by treatment with 10 mM citrate buffer for 5 min at 121 °C using an autoclave. For immunoperoxidase staining, tissue sections were incubated with anti-milk (1:2 × 10^5^) antibody and treated with Signal Stain Boost IHC Detection Reagent (Cell Signaling Technology). Horseradish peroxidase signals were detected using a HistoMark ORANGE kit (KPL) according to the manufacturer’s protocol. Nuclei were stained with hematoxylin. For immunofluorescence staining, sections were incubated with anti-Krt5 (1:200), anti-E-cad (1:200), and anti-cyclin D1 (1:200) primary antibodies, and detected with anti-mouse-Alexa488 (1:100) and anti-rabbit-Alexa546 (1:100) secondary antibodies. Cell nuclei were stained with 1 μg mL^−1^ Hoechst 33342 (#080-09981, Wako). Fluorescent signals were detected using an FW1000 confocal microscope (Olympus). For immunofluorescence staining of spheres, they were embedded in iPGell (GenoStaff, Tokyo, Japan) and fixed with 4% paraformaldehyde for 1 h according to the manufacturer’s protocol. Gels containing spheres were embedded in paraffin and sectioned in 5 μm slices. TUNEL staining of mammary glands was performed using an In situ Apoptosis Detection Kit (Takara) according to the manufacturer’s protocol. After TUNEL staining, sections were incubated with anti-E-cad (1:200) primary antibody and detected with anti-mouse-Alexa546 (1:100) secondary antibody. Cell nuclei were stained with 1 μg mL^−1^ Hoechst 33342.

### In vitro mammosphere formation assay

Cells sorted using flow cytometry were resuspended at densities of 1000–30 cells per well for cells isolated from recipient mice in the virgin stage, 1000–31 cells per well for luminal cells, and 700–20 cells per well for basal cells isolated from 10-day-old female mice in chilled EpiCult-B mouse medium supplemented with 10 ng mL^−1^ EGF, 10 ng mL^−1^ bFGF, 5 μg mL^−1^ insulin, 1 μg mL^−1^ hydrocortisone, and 4 μg mL^−1^ heparin. Next, the cells were mixed in 5% growth-factor-reduced Matrigel. The number and size of spheres (> 50 μm) were scored after 16 days of culture. Sphere-forming units were calculated as previously described^62^.

### Statistical analysis and reproducibility

Statistically significant differences between the mean values were determined using two-tailed Student’s *t*-tests. All data are representative of three independent experiments. The values represent the means ± SD and *p*-values < 0.05 were considered statistically significant. Exact *p*-values are presented in Supplementary Table 3.

### Reporting summary

Further information on research design is available in the Nature Research Reporting Summary linked to this article.

## Supplementary information

Supplementary Information

Description of Additional Supplementary Files

Supplementary Data 1

Reporting Summary

## Data Availability

The data that support the findings of this study are available in Supplementary Data 1 and from the authors on reasonable request.
