# Supplementary material for: TRAF6 maintains mammary stem cells and promotes pregnancy-induced mammary epithelial cell expansion
Source: Commun Biol. 2019 Aug 6;2:292. doi: 10.1038/s42003-019-0547-7 (PMC6684589; doi:10.1038/s42003-019-0547-7)
Supplement: Supplementary file 1 — Supplementary Information [file 42003_2019_547_MOESM1_ESM.pdf]

Supplementary Figures

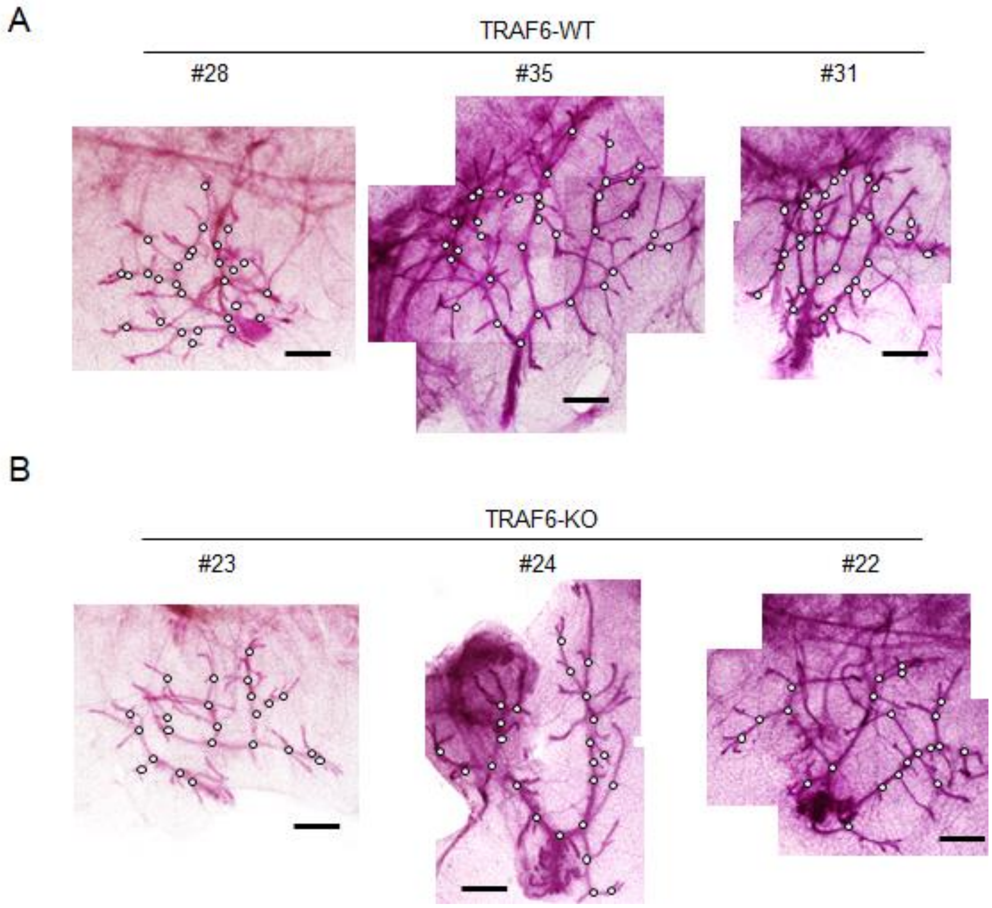

Supplementary Fig. 1. TRAF6 is partially involved in branching of mammary ducts but not essential for mammary gland development during prepuberty.

Representable images of whole-mount analysis of mammary tissue of 10-day-old TRAF6-WT (A) and TRAF6-KO (B) female mice. Fat pads were removed and placed on a microscope slide followed by staining in a carmine-Alum mix. Branching points were carefully marked under the microscope with specific attention to discrimination between mammary ducts and other tubes including blood and lymphatic vessels and to discrimination between branch and spatial contiguity. White circles indicate branching points. The numerical values indicate number of branching points. Scale bars, 500 μm.

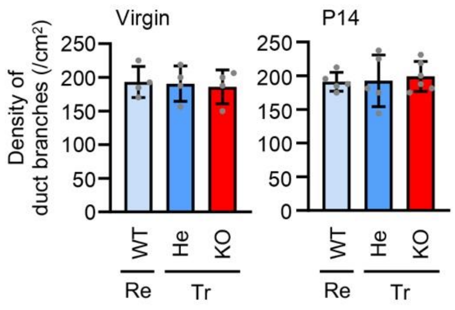

Supplementary Fig. 2. TRAF6 is not essential for side branching in mammary gland in virgin and P14 stages.

Branching morphogenesis in the mammary gland in #2 fat pads of recipient wild type mice and the outgrowths derived from transplanted TRAF6-He and TRAF6-KO epithelia in cleared fat pads at the virgin (8 weeks after transplantation) and P14 stages. Values are means ± s.d. (virgin, n = 4; P14, n = 5).

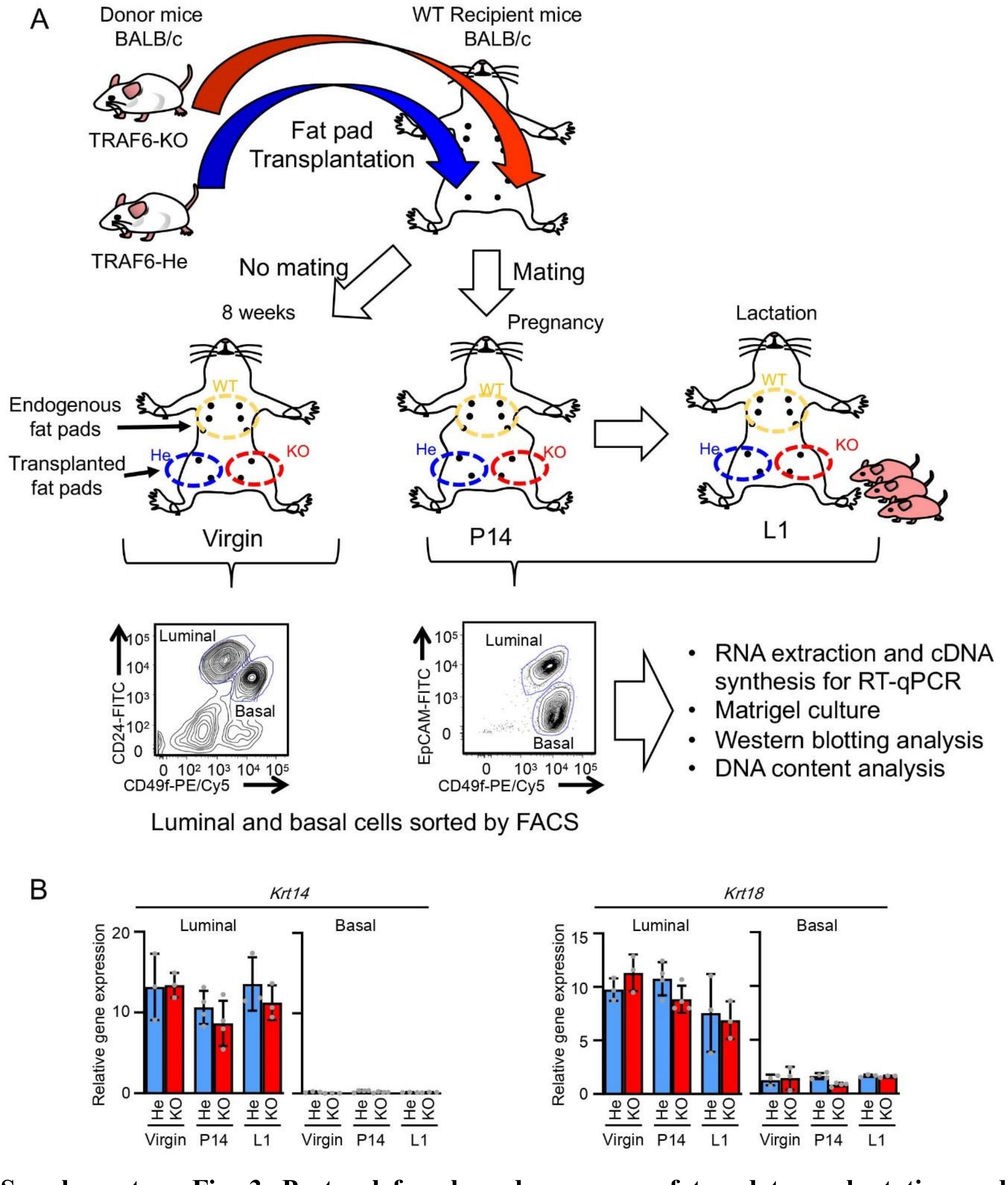

Supplementary Fig. 3. Protocol for cleared mammary fat pad transplantation and mammary epithelial cell sorting.

(**A**) Schematic representation of the protocol for cleared mammary fat pad transplantation and mammary epithelial cell sorting with subsequent analyses. (**B**) Real-time RT-qPCR

analysis of *Krt14* and *Krt18* expression in luminal and basal cells sorted from outgrowths developed from transplanted TRAF6-He and TRAF6-KO epithelia in recipient mice at the virgin, P14, and L1 stages. Values are means ± s.d. (Virgin, n = 3; P14, n = 4; L1, n = 3).

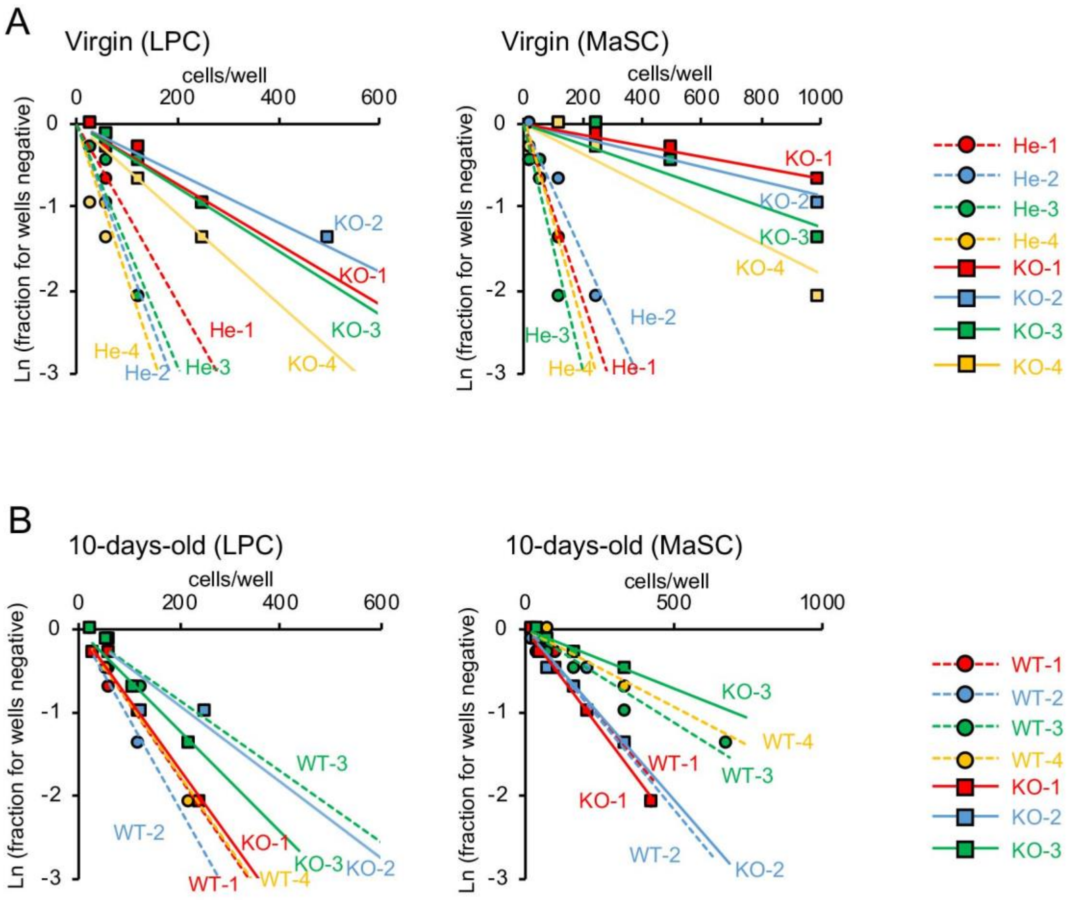

Supplementary Fig. 4. TRAF6 is required for maintaining mammary stem cells (MaSCs) and luminal progenitor cells (LPCs).

(**A**) *In vitro* limiting-dilution assay of luminal and basal cells isolated from outgrowths of transplanted TRAF6-He and TRAF6-KO epithelia in recipient mice in the virgin stage. Isolated cells were cultured in ultra-low adherent plates at densities of 1000 to 31 cells/well. At 16 days after seeding, spheres (>50 μm) were counted to calculate the sphere forming unit. Each line is constructed using the data points obtained from at least three cell densities for all individuals tested. (TRAF6-He and TRAF6-KO, n = 4/group). (**B**) *In vitro* limiting-dilution assay of luminal and basal cells isolated from mammary tissue of 10-day-old (prepubertal) TRAF6-WT and TRAF6-KO female mice. Isolated cells were cultured in ultra-low adherent plates at densities of 1000 to 30 cells/well for luminal cells and 700 to 20 cells/well for basal cells. At 16 days after seeding, spheres (>50 μm) were counted to calculate the sphere forming unit. Each line is constructed using the data points obtained from

at least three cell densities for all individuals tested. (TRAF6-WT, n=4; TRAF6-KO, n = 3). Ln, natural logarithm.

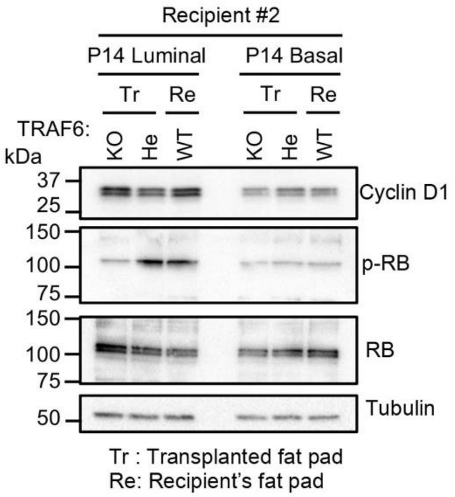

Supplementary Fig. 5. TRAF6 is required for Rb phosphorylation in luminal cells during pregnancy.

Western blotting analysis of Cyclin D1, pRB, and RB in luminal and basal cells isolated from outgrowths in recipient mice (#2) at P14. Tr: Transplanted fat pad; Re: Recipient's fat pad.

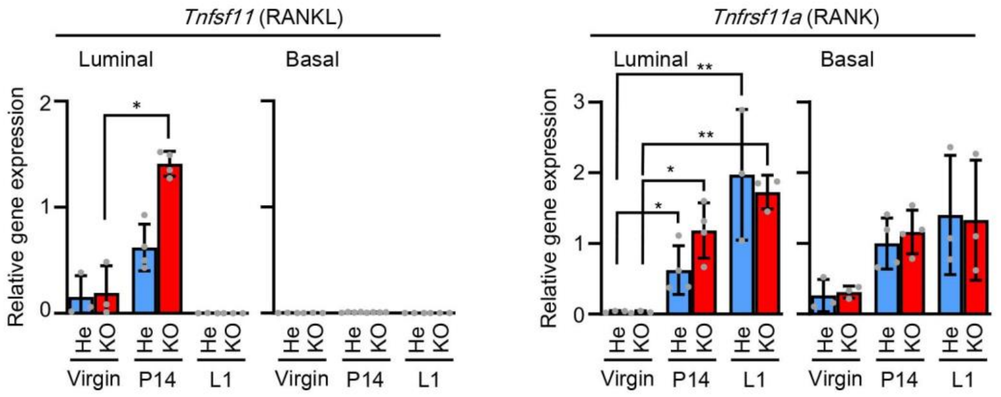

Supplementary Fig. 6. TRAF6-deficiency does not affect RANKL (*Tnfsf11*) and RANK (*Tnfrsf11a*) expression in mammary epithelial cells.

Real-time RT-qPCR analysis of *Tnfsf11* and *Tnfrsf11a* expression in luminal and basal cells isolated from outgrowths developed from TRAF6-He and TRAF6- KO epithelia in recipient mice at the virgin, P14, and L1 stages. Values are mean ± s.d. (Virgin, n = 3; P14, n = 4; L1, n = 3; *p < 0.05 and **p < 0.01).

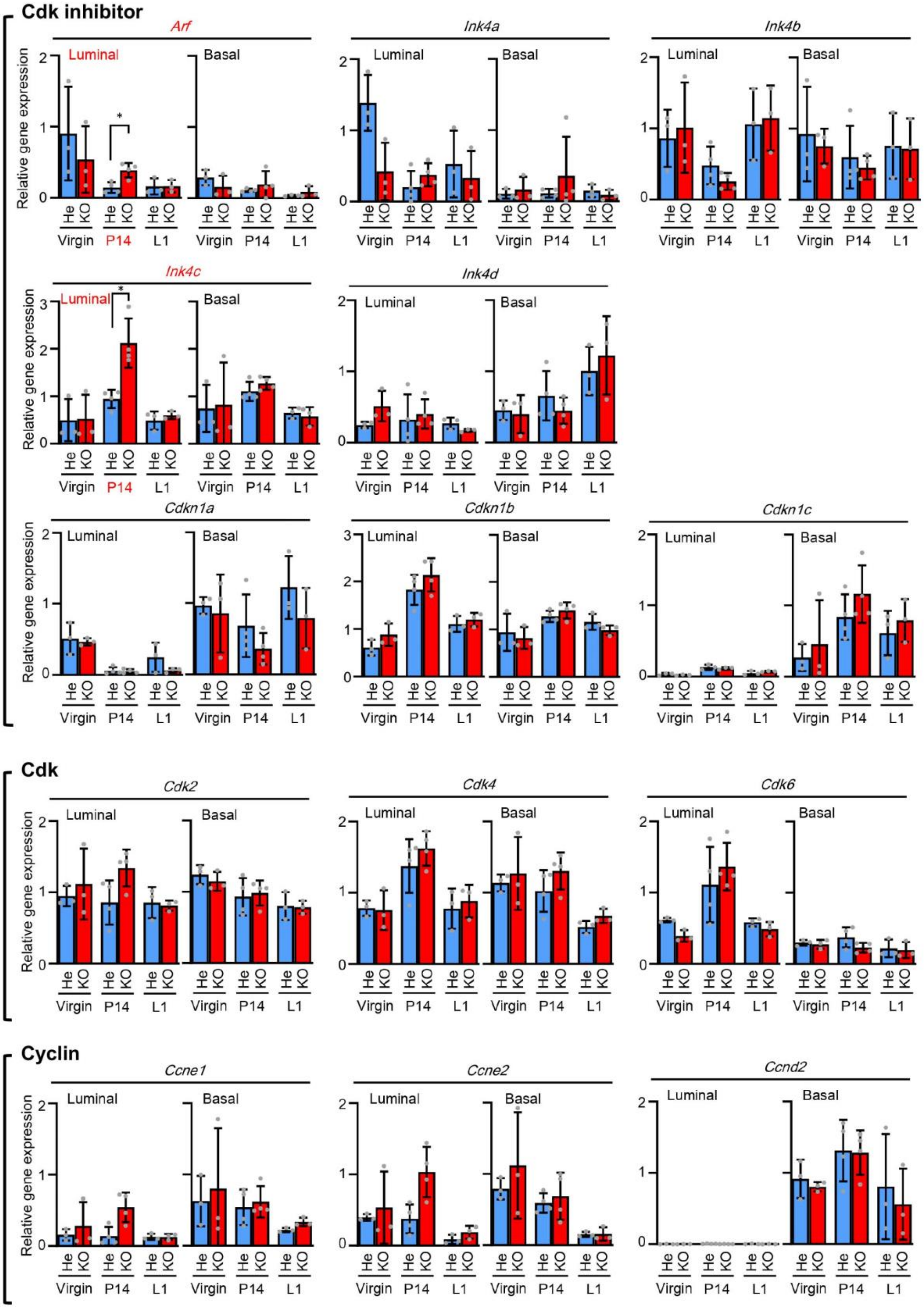
Cdk inhibitor
Arf
Ink4a
Ink4b
Ink4c
Ink4d
Cdkn1a
Cdkn1b
Cdkn1c
Cdk
Cdk2
Cdk4
Cdk6
Cyclin
Ccne1
Ccne2
Ccnd2
Luminal
Basal
Relative gene expression
He
KO
Virgin
P14
L1
*

Supplementary Fig. 7. *Arf* and *Ink4c* expression is significantly upregulated at P14 TRAF6-KO luminal cells.

Real-time RT-qPCR analysis of mRNA expression of various cell proliferation-related genes in luminal and basal cells isolated from outgrowths developed from TRAF6-He and TRAF6-KO epithelia in recipient mice at the virgin, P14, and L1 stages. Values are means ± s.d. (Virgin, n = 3; P14, n = 4; L1, n = 3; *$p < 0.05$).

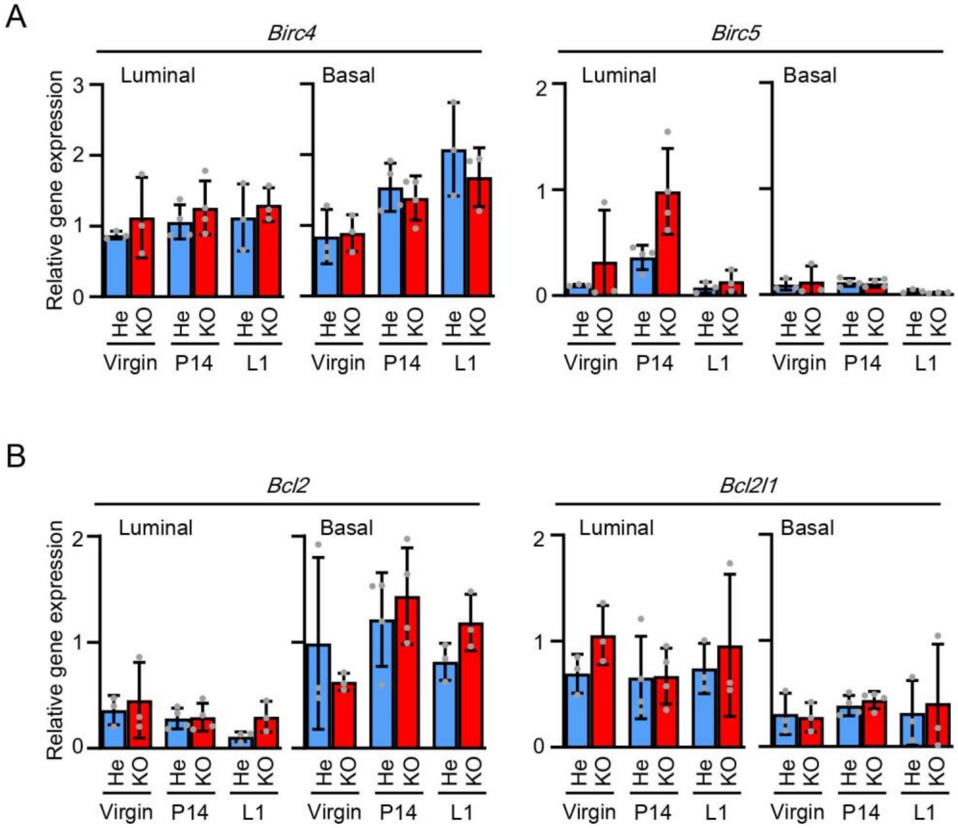

Supplementary Fig. 8. TRAF6 deficiency does not affect expression of some anti-apoptotic gene expression.

Real-time RT-qPCR analysis of *Birc4 and Birc5* expression (**A**) and that of *Bcl2* and *Bcl2l1* expression (**B**) in luminal and basal cells isolated from outgrowths developed from TRAF6-He and TRAF6-KO epithelia in recipient mice at the virgin, P14, and L1 stages. Values are means ± s.d. (Virgin, n = 3; P14, n = 4; L1, n = 3).

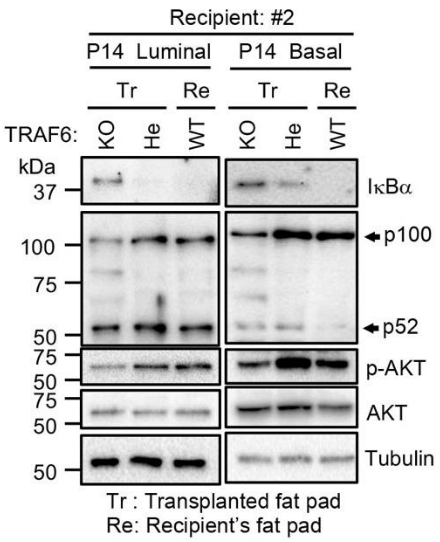

Supplementary Fig. 9. TRAF6 selectively activates canonical NF-κB and AKT pathways in mammary epithelial cells during pregnancy.

Western blotting analysis of IκBα, p100, p52, phosphorylated AKT (p-AKT), and AKT expression in luminal and basal cells isolated from outgrowths in recipient mice (#2) at P14. Tr: Transplanted fat pad; Re: Recipient's fat pad.

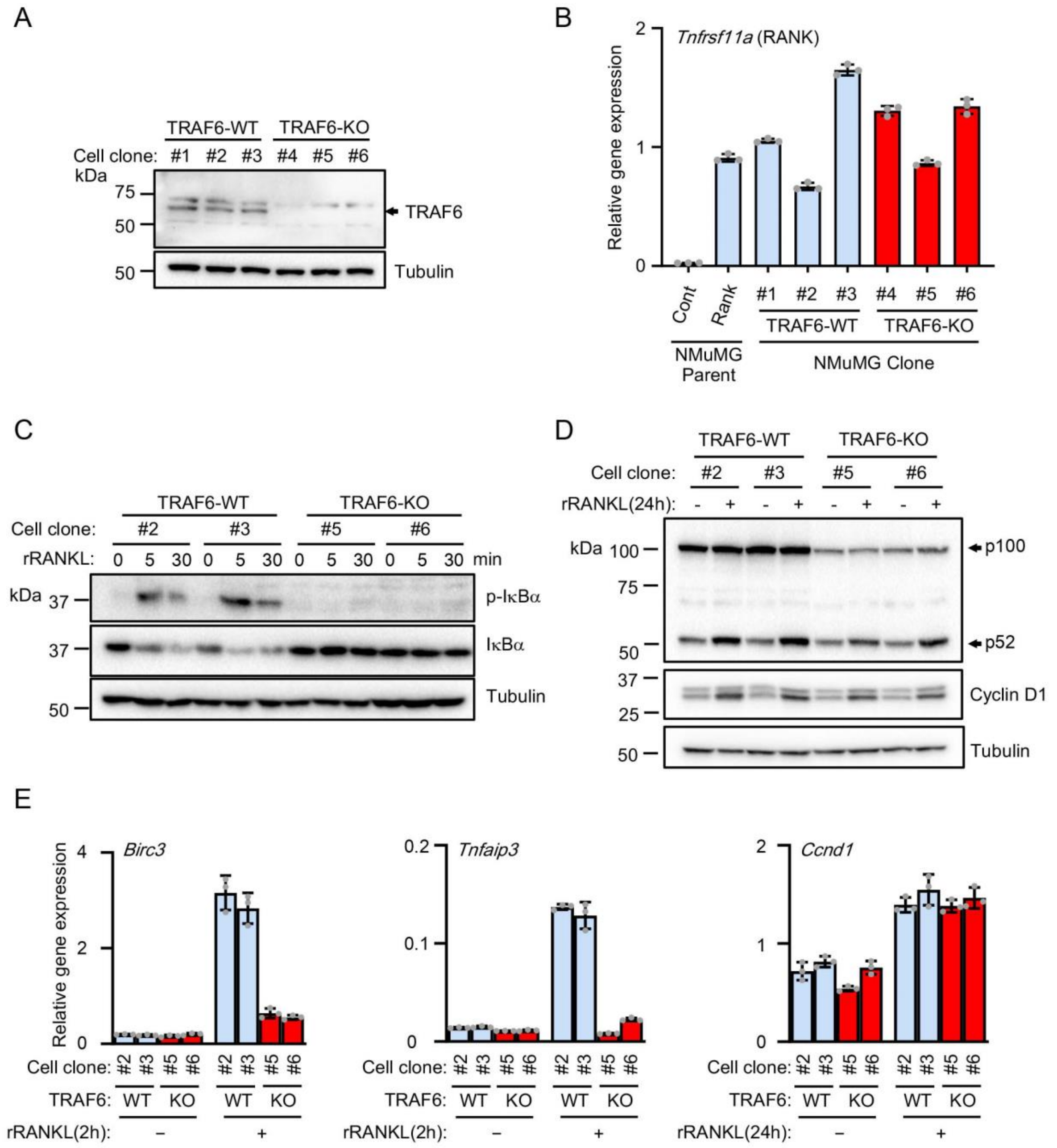

Supplementary Fig. 10. RANK-induced canonical and noncanonical NF-κB pathways have distinct roles in the pregnancy-dependent expansion of mammary epithelial cells. (**A**) Western blotting analysis of TRAF6 expression in TRAF6-WT or TRAF6-KO NMuMG-RANK clones. Arrow indicates endogenous TRAF6. (**B**) Real-time RT-qPCR analysis of *Tnfrsf11a* (RANK) expression in NMuMG-RANK clones. Values are means ± s.d. (n = 3).

(C) Western blotting analysis of p-IκBα and IκBα in NMuMG-RANK clones #2 and #3 (TRAF6-WT) and #5 and #6 (TRAF6-KO). Cells were pre-treated with serum-reduced DMEM (1% FBS) for 8 h and then stimulated with rRANKL for the indicated time. **(D)** Western blotting analysis of p100, p52, and Cyclin D1 expression in NMuMG-RANK clones #2 and #3 (TRAF6-WT) and #5 and #6 (TRAF6-KO). Cells were pre-treated as in (**C**) and then stimulated with rRANKL for 24 h. **(E)** Real-time RT-qPCR analysis of *Birc3*, *Tnfaip3*, and *Ccnd1* expression in TRAF6-WT or TRAF6-KO NMuMG-RANK clones. Total RNA was prepared and subjected to RT-qPCR. Values are means ± s.d. ($n = 3$; $^{**}p < 0.01$).

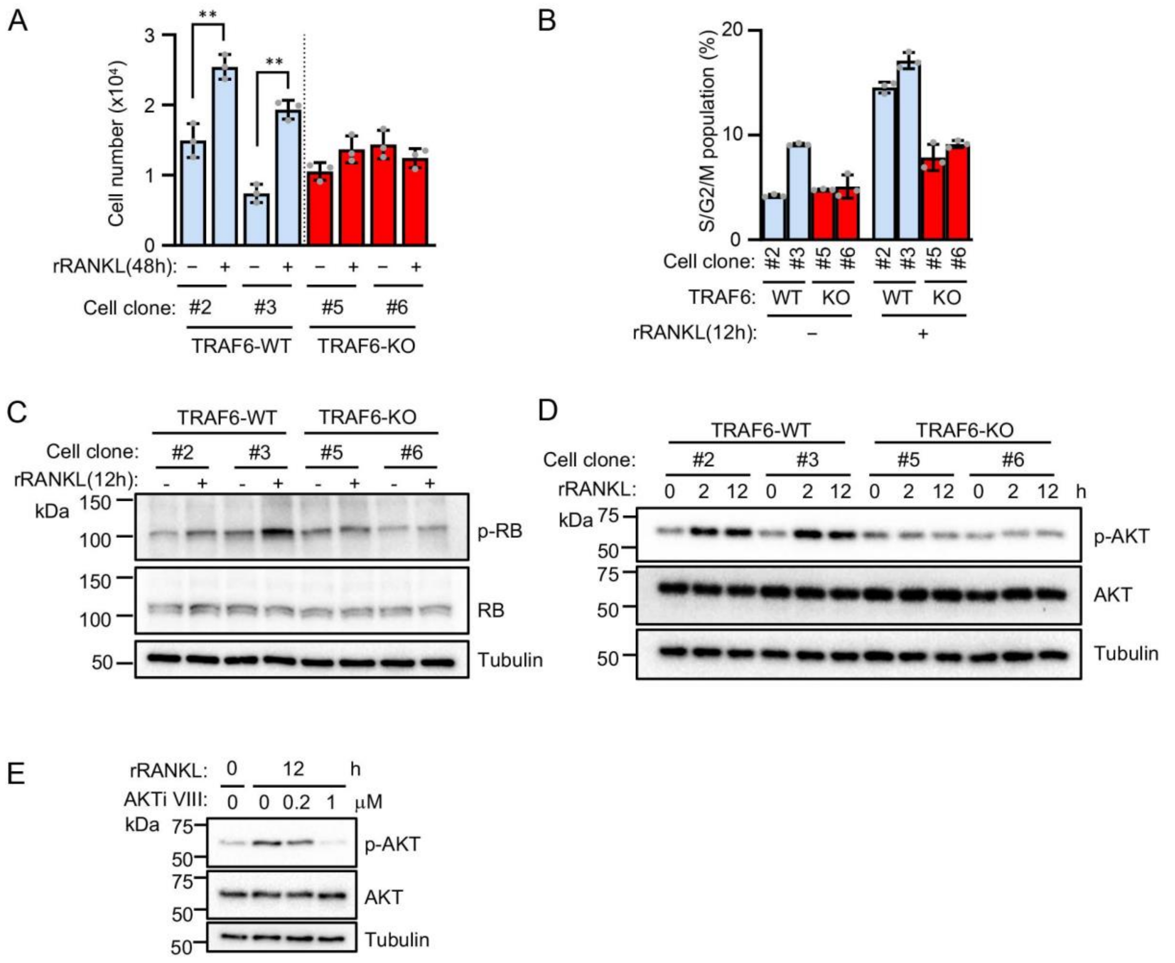

Supplementary Fig. 11. Cell proliferation following RANKL stimulation is induced by TRAF6-dependent activation of AKT leading to Rb phosphorylation.

(**A**) RANK-TRAF6 pathway-dependent proliferation of NMuMG-RANK clones #2 and #3 (TRAF6-WT) and #5 and #6 (TRAF6-KO). Cells were pre-treated with serum-reduced DMEM (1% FBS) for 8 h and then either untreated or stimulated with RANKL for 48 h. Cell number was then counted. Values are means ± s.d. ($n = 3$, *$p < 0.05$ and **$p < 0.01$). (**B**) S/G2/M phase population was analyzed in TRAF6-WT or TRAF6-KO NMuMG-RANK clones after RANKL stimulation. Cells were pre-treated as in (**A**) and then stimulated with RANKL for 12 h. Values are means ± s.d. ($n = 3$, **$p < 0.01$). **(C)** Western blotting analysis of RB phosphorylation in TRAF6-WT or TRAF6-KO NMuMG-RANK clones after RANKL stimulation. Cells were treated as in (**B**). (**D**) Western blotting analysis of AKT phosphorylation in TRAF6-WT or TRAF6-KO NMuMG-RANK clones after RANKL

stimulation. Cells were pre-treated with serum-reduced DMEM (1% FBS) for 8 h and then stimulated with RANKL for the indicated time. (**E**) Western blotting analysis of AKT phosphorylation in AKT inhibitor-treated NMuMG-RANK cells after RANKL stimulation. Cells were pre-treated as in (**A**) and then treated with different concentrations of AKTi VIII for 60 min followed by RANKL stimulation with AKTi VIII.

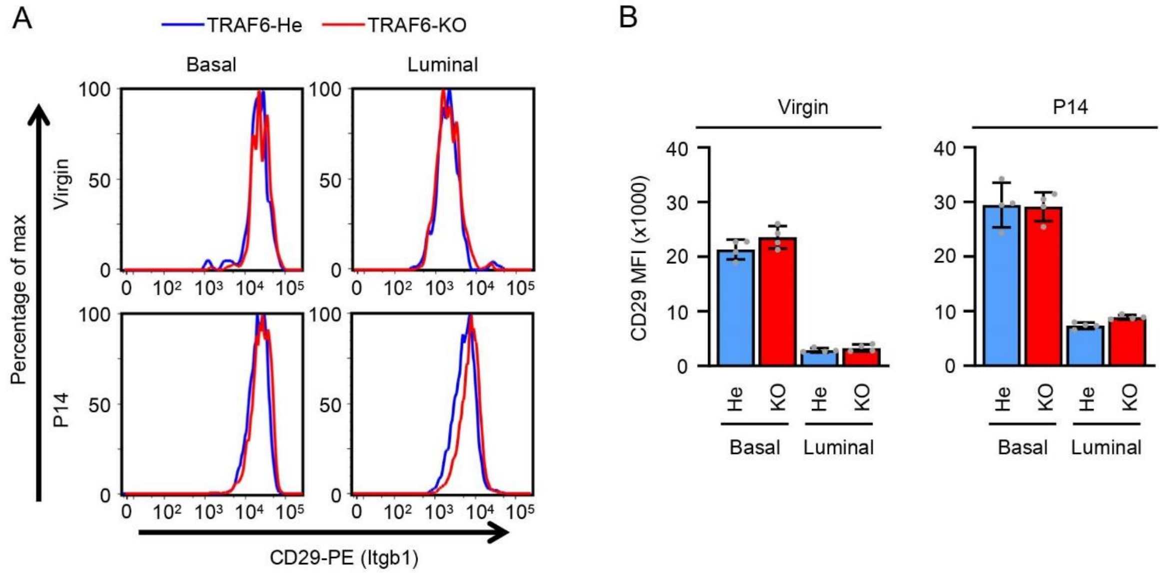

Supplementary Fig. 12. Integrin β1 (CD29) expression in epithelial cells at the virgin stage and pregnancy day 14 (P14) is normal in TRAF6-KO glands (**A**) Flow cytometric analysis of mammary epithelial cells isolated from outgrowths developed from TRAF6-He (blue lines) and TRAF6-KO (red lines) epithelia from virgin and P14 recipient mice. Cells in virgin recipients were stained for CD24, CD49f, and CD29 and those in P14 recipients were stained for EpCAM, CD49f, and CD29. CD29 expression in $CD24^{high}CD49f^{mid}$ luminal cells (upper right panel) and $CD24^{mid}CD49f^{high}$ basal cells (upper left panel) from virgin recipients. CD29 expression in $EpCAM^{high}CD49f^{mid}$ luminal cells (lower right panel) and $EpCAM^{mid}CD49f^{high}$ basal cells (lower left panel) from P14 recipients. (**B**) Mean fluorescence intensities of CD29 expression determined using flow cytometric analyses in (**A**). Values are means ± s.d. (Virgin and P14, n = 3/group).

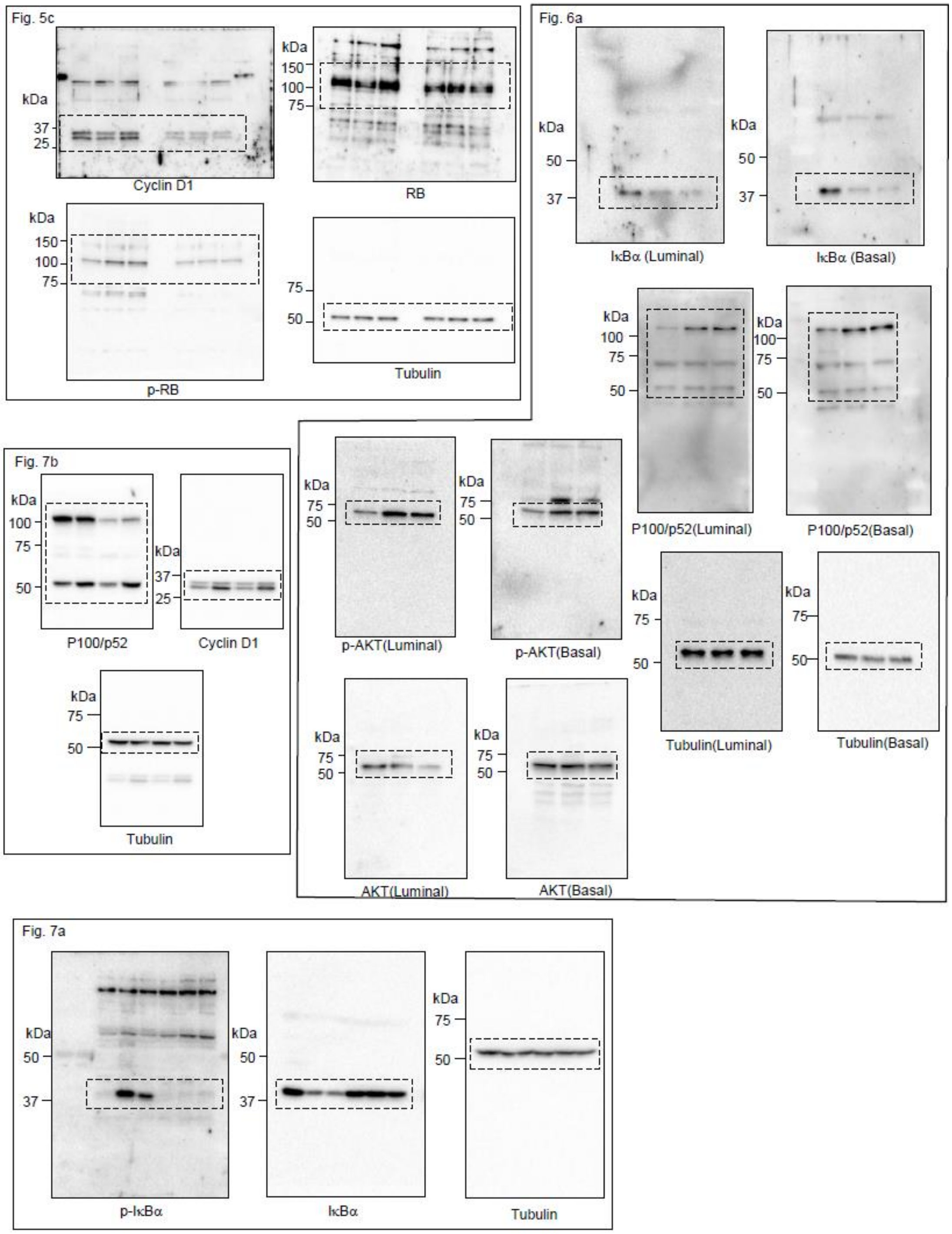

Supplementary Fig. 13. Full-scan images of immunoblots. Dotted boxes indicate cropped images used in the figures.

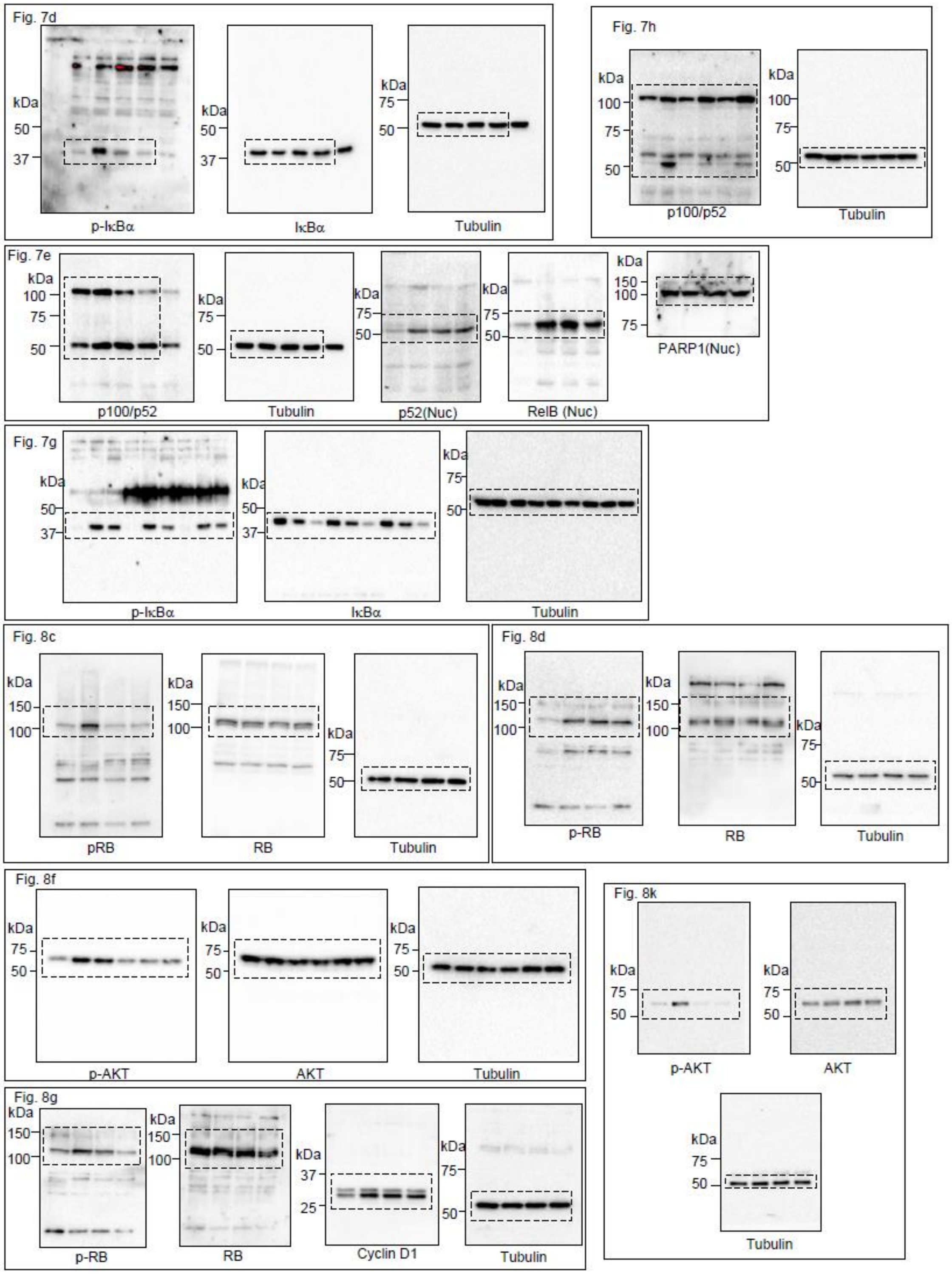

Supplementary Fig. 13. Continued

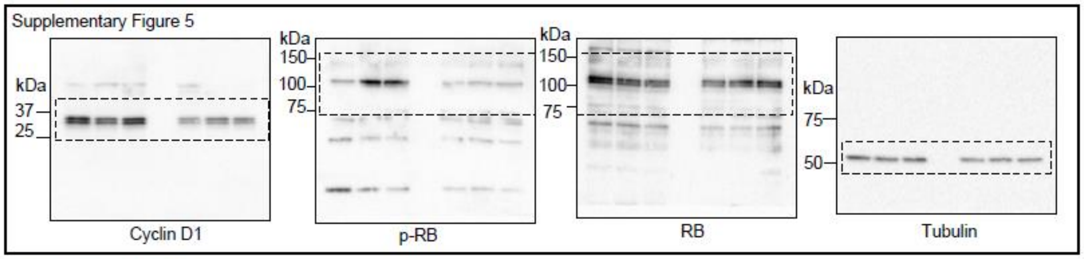

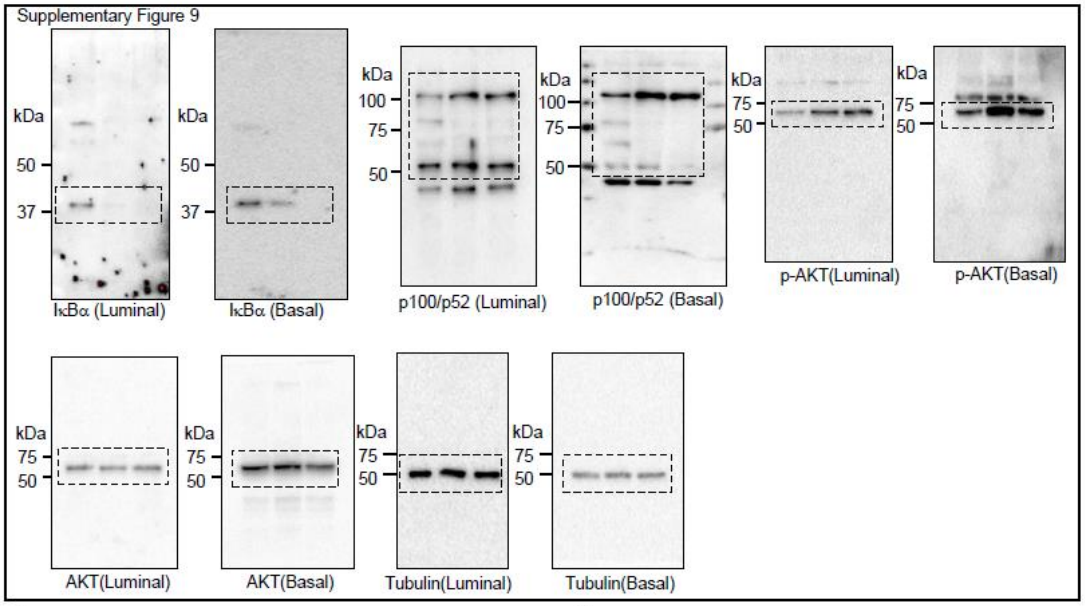

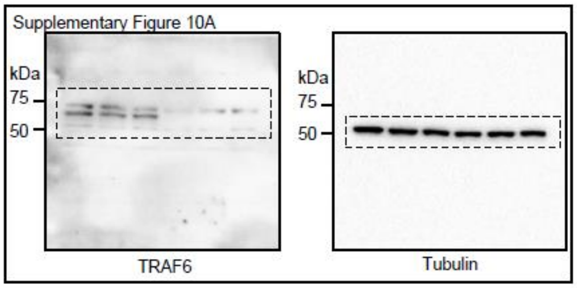

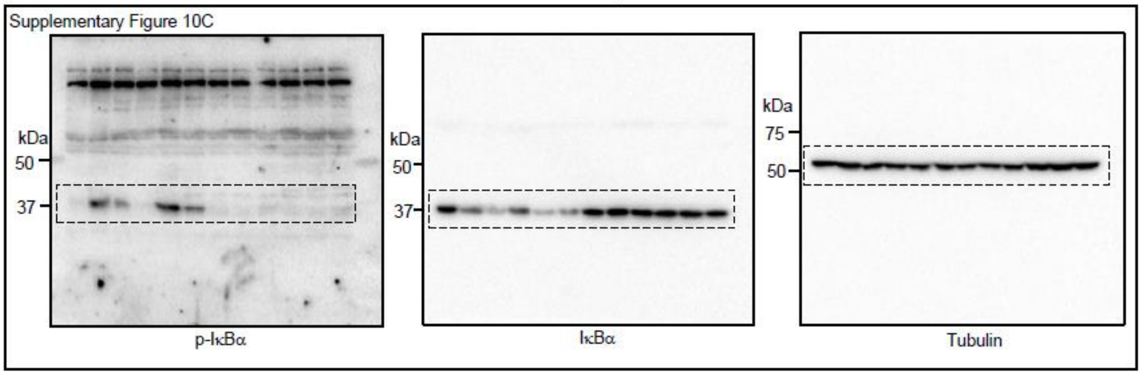

Supplementary Fig. 13. Continued

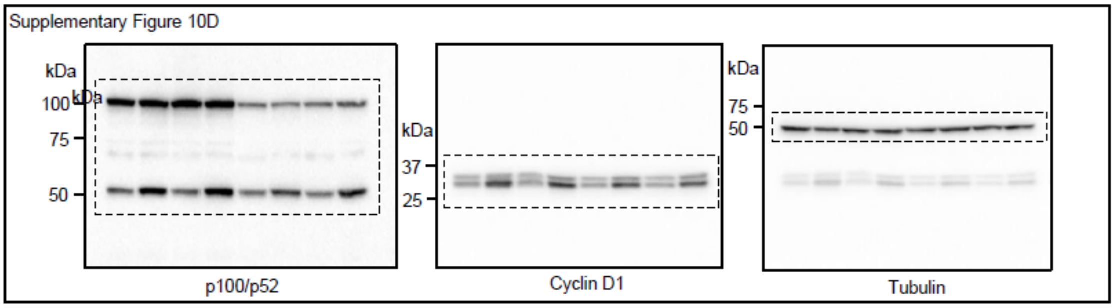

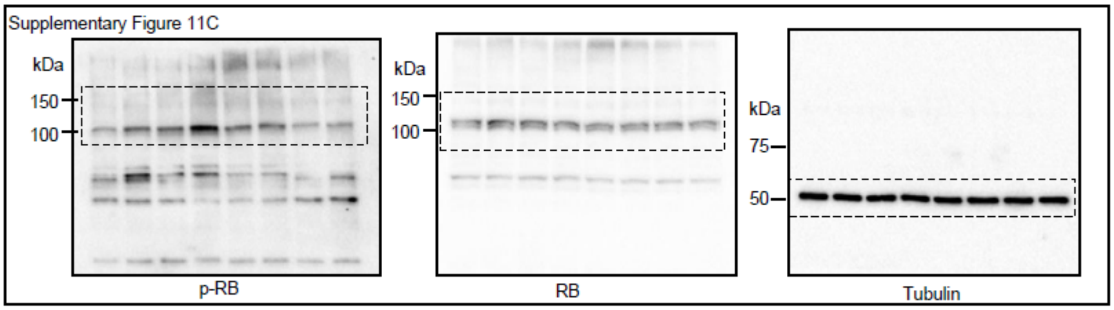

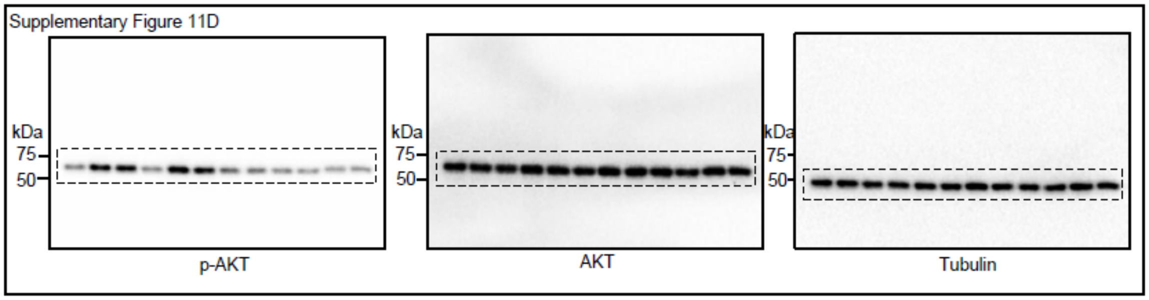

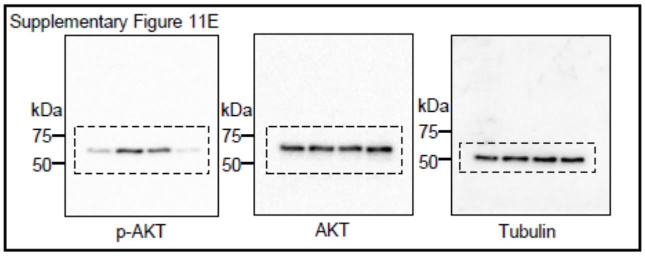

Supplementary Fig. 13. Continued

Supplementary Tables

Supplementary Table 1. Oligonucleotides used for RNAi experiments,

Map3k14 stealth RNA#1	GGGUCCUGCUUACUGAGAAACUCAA
Map3k14 stealth RNA#2	GACAGGCUUCCAGUGUGCUGUCAAA

Supplementary Table 2. Primer sets for real time RT-PCR.

Actb	GGCTGTATTCCCCTCCATCG
	CCAGTTGGTAACAATGCCATG
Traf6	AAGCCTGCATCATCAAATCC
	ATTTGGGCACTTTACCGTCA
Krt14	GTCGATCTGCAGGAGGACAT
	GAGGTGAAGATTCGGGACTG
Krt18	GACTCTAAAGTCATCGGCGG
	TCAGAGACTGGGGCCACTAC
Csn2	ACATTTACTGTATCCTCTGAGACTG
	TAGCCTGGAGCACATCCTCT
Wap	GACTTGGGCTGGTCACTCC
	GACCGTGAGTGTTCTGGCAA
Ccnd1	GCGTACCCTGACACCAATCT
	CTCTTCGCACTTCTGCTCCT
Birc2	TGGCCACTGGTGAGAACTAC
	GAGGGCCATTCTATTCTTCCGA
Birc3	CCCGGAGATCAGAGGTCATTG
	AAGGCGCTGTCTTGAACCAT
Birc4	AATCCAAACATCCGGGAGCA
	TCTTGTAGGCGCCTTAGCTG
Birc5	GAACCCGATGACAACCCGAT
	TGGTCTCCTTTGCAATTTTGTTCT
Bcl2l1	AGAAGAAACTGAAGCAGAG
	TCCGACTCACCAATACCTG
Bcl2	GAG GAT TGT GGC CTT CTT TG
	CGT TAT CCT GGA TCC AGG TG
Tnfaip3	ACAGTGGACCTGAACTTCGC
	TTGATCAGGTGAGTCGTGCC

Tnfsf11	GTTGCTTAACGTCATGTTAGA
	CTGAGGCCCAGCCATTTG
Tnfrsf11a	GCTGGCTACCACTGGAACTC
	GTGCAGTTGGTCCAAGGTTT
Nfkbia	TGAAGGACGAGGAGTACGAGC
	TTCGTGGATGATTGCCAAGTG
Cdk2	GTACCCAGTACTGCCATCCG
	GGTGAAGGACACGGTGAGAA
Cdk4	TTTGCAGAGATGTTCCGTCG
	GCAGAGATTCGCTTATGTGGG
Cdk6	TCAGCAGTATGAGTGCGTGG
	GTTTCTCTGTCCGTCCGTGA
Ccne1	GGCAAATGTGGCCGTGTTTT
	ATTCAAGACGGGAAGTGGGG
Ccne2	TGCTGCCGCCTTATGTCATTT
	GACAGCTGCCCTCCTTTTCTG
Ink4a	CGAACTCGAGGAGAGCCATC
	TACGTGAACGTTGCCCATCA
Arf	TGGTCACTGTGAGGATTCAGC
	CGTGAACGTTGCCCATCATC
Ink4b	CAGATCCCAACGCCCTGAAC
	CACAGGTCTGGTAAGGGTGG
Ink4c	GGGGGACCTAGAGCAACTTA
	GGAGAAGCCTCCTGGCAATC
Ink4d	AGGAAAGGAGGGAGGTCCAC
	ACTGCTGGACTTCCAAACATCA
Cdkn1a	GTACTTCCTCTGCCCTGCTG
	TCTGCGCTTGGAGTGATAGA
Cdkn1b	TTGGGTCTCAGGCAAACTCT

	TCTGTTCTGTTGGCCCTTTT
Cdkn1c	CGGCCAATGCGAACGACTT
	CAGGAGCCACGTTTGGAGAG
Rpl13a	CCCTCCACCCTATGACAAGA
	GCCCCAGGTAAGCAAACTT

Supplementary Table 3. Exact p-values.

			p-value(T.TEST)
Fig.1	(b)	WT vs KO	p = 3.05x10^-5
Fig.3	(a)	Luminal progenitor	p = 4.70x10^-3
		MaSC	p = 9.66x10^-4
Fig.4	(a)	Luminal Virgin vs P14	p = 3.58x10^-2
		Luminal Virgin vs L1	p = 4.14x10^-2
Fig.5	(a)	Ccnd1 He Virgin vs P14	p = 4.93x10^-2
		Ccnd1 KO Virgin vs P14	p = 3.74x10^-2
	(d)	Luminal WT vs KO	p = 2.50x10^-2
		Luminal He vs KO	p = 4.99x10^-3
		Basal WT vs KO	p = 4.41x10^-2
		Basal He vs KO	p = 2.00x10^-3
	(e)	Birc2 Luminal P14 He vs KO	p = 1.19x10^-2
		Birc2 Luminal L1 He vs KO	p = 4.02x10-^4
		Birc3 Luminal P14 He vs KO	p = 2.38x10^-2
		Birc3 Luminal L1 He vs KO	p = 6.86x10^-3
		Tnfaip3 Luminal P14 He vs KO	p = 7.15x10^-3
		Tnfaip3 Luminal L1 He vs KO	p = 3.47x10^-2
		Tnfaip3 Basal L1 He vs KO	p = 3.39x10^-4
	(g)	P14 He vs KO	p = 1.58x10^-4
		L1 He vs KO	p = 6.18x10^-3
Fig.6	(b)	Virgin Luminal He vs KO	p = 1.51x10^-2
		P14 Luminal He vs KO	p = 2.18x10^-5
		L1 Luminal He vs KO	p = 1.30x10^-3
		P14 Basal He vs KO	p = 7.16x10^-5
		L1 Basal He vs KO	p = 2.15x10^-2
Fig.7	(c)	Birc3 2 h WT vs KO	p = 4.45x10^-5
		Tnfaip3 2 h WT vs KO	p = 7.37x10^-7
	(f)	Birc3 TPCA1 0 vs 0.3 uM	p = 2.66x10^-2
		Tnfaip3 TPCA1 0 vs 0.3 uM	p = 2.01x10^-3

	(i)	Ccnd1 Rankl 2 h siCont vs siNIK#1	p = 1.55x10^-2
		Ccnd1 Rankl 2 h siCont vs siNIK#2	p = 8.10x10^-3
Fig.8	(a)	WT 0 h vs 48 h	p = 1.08x10^-2
		KO 0 h vs 48 h	p = 4.86x10^-2
	(b)	Rankl WT vs KO	p = 1.33x10^-4
	(h)	Rankl DMSO vs AKTi	p = 8.73x10^-6
	(i)	He Cont vs Rankl	p = 2.44x10^-2
	(j)	Rankl DMSO vs AKTi	p = 1.11x10^-2
