# Supplementary material for: TRAF6 maintains mammary stem cells and promotes pregnancy-induced mammary epithelial cell expansion
Source: Commun Biol. 2019 Aug 6;2:292. doi: 10.1038/s42003-019-0547-7 (PMC6684589; doi:10.1038/s42003-019-0547-7)
Supplement: Supplementary file 2 — Description of Additional Supplementary Files [file 42003_2019_547_MOESM2_ESM.pdf]

Descriptions of additional supplementary files

Name: Supplementary Data.xlsx

Description: The Supplementary Data file contains all source data underlying the graphs presented in the main figures (Figure 1- 8).
