# Supplementary material for: TRAF6 maintains mammary stem cells and promotes pregnancy-induced mammary epithelial cell expansion
Source: Commun Biol. 2019 Aug 6;2:292. doi: 10.1038/s42003-019-0547-7 (PMC6684589; doi:10.1038/s42003-019-0547-7)
Supplement: Supplementary file 4 — Reporting Summary [file 42003_2019_547_MOESM4_ESM.pdf]

nature research

Corresponding author(s): Jun-ichiro Inoue

Last updated by author(s): Jul 8, 2019

# Reporting Summary

Nature Research wishes to improve the reproducibility of the work that we publish. This form provides structure for consistency and transparency in reporting. For further information on Nature Research policies, see Authors & Referees and the Editorial Policy Checklist.

## Statistics

For all statistical analyses, confirm that the following items are present in the figure legend, table legend, main text, or Methods section.

| n/a | Confirmed | |
|---|---|---|
| ☐ | ☒ | The exact sample size ($n$) for each experimental group/condition, given as a discrete number and unit of measurement |
| ☐ | ☒ | A statement on whether measurements were taken from distinct samples or whether the same sample was measured repeatedly |
| ☐ | ☒ | The statistical test(s) used AND whether they are one- or two-sided<br>*Only common tests should be described solely by name; describe more complex techniques in the Methods section.* |
| ☒ | ☐ | A description of all covariates tested |
| ☒ | ☐ | A description of any assumptions or corrections, such as tests of normality and adjustment for multiple comparisons |
| ☐ | ☒ | A full description of the statistical parameters including central tendency (e.g. means) or other basic estimates (e.g. regression coefficient) AND variation (e.g. standard deviation) or associated estimates of uncertainty (e.g. confidence intervals) |
| ☐ | ☒ | For null hypothesis testing, the test statistic (e.g. $F$, $t$, $r$) with confidence intervals, effect sizes, degrees of freedom and $P$ value noted<br>*Give P values as exact values whenever suitable.* |
| ☒ | ☐ | For Bayesian analysis, information on the choice of priors and Markov chain Monte Carlo settings |
| ☒ | ☐ | For hierarchical and complex designs, identification of the appropriate level for tests and full reporting of outcomes |
| ☒ | ☐ | Estimates of effect sizes (e.g. Cohen's $d$, Pearson's $r$), indicating how they were calculated |

*Our web collection on statistics for biologists contains articles on many of the points above.*

## Software and code

Policy information about availability of computer code

| | |
|---|---|
| Data collection | n/a |
| Data analysis | n/a |

For manuscripts utilizing custom algorithms or software that are central to the research but not yet described in published literature, software must be made available to editors/reviewers. We strongly encourage code deposition in a community repository (e.g. GitHub). See the Nature Research guidelines for submitting code & software for further information.

## Data

Policy information about availability of data

All manuscripts must include a data availability statement. This statement should provide the following information, where applicable:

- Accession codes, unique identifiers, or web links for publicly available datasets
- A list of figures that have associated raw data
- A description of any restrictions on data availability

The data that support the findings of this study are available from the authors on reasonable request.

# Field-specific reporting

Please select the one below that is the best fit for your research. If you are not sure, read the appropriate sections before making your selection.

☒ Life sciences ☐ Behavioural & social sciences ☐ Ecological, evolutionary & environmental sciences

For a reference copy of the document with all sections, see nature.com/documents/nr-reporting-summary-flat.pdf

# Life sciences study design

All studies must disclose on these points even when the disclosure is negative.

| | |
|---|---|
| Sample size | For all experiments, more than three samples were allocated into each experimental groups to determine standard deviation and statistically significant differences using two-tailed Student's t-tests. |
| Data exclusions | No data were excluded. |
| Replication | All our findings were successfully confirmed by three independent experiments. |
| Randomization | All cell samples and mice were randomly allocated into experimental groups. |
| Blinding | n/a |

# Reporting for specific materials, systems and methods

We require information from authors about some types of materials, experimental systems and methods used in many studies. Here, indicate whether each material, system or method listed is relevant to your study. If you are not sure if a list item applies to your research, read the appropriate section before selecting a response.

## Materials & experimental systems

| n/a | Involved in the study | |
|---|---|---|
| ☐ | ☒ | Antibodies |
| ☐ | ☒ | Eukaryotic cell lines |
| ☒ | ☐ | Palaeontology |
| ☐ | ☒ | Animals and other organisms |
| ☒ | ☐ | Human research participants |
| ☒ | ☐ | Clinical data |

## Methods

| n/a | Involved in the study | |
|---|---|---|
| ☒ | ☐ | ChIP-seq |
| ☐ | ☒ | Flow cytometry |
| ☒ | ☐ | MRI-based neuroimaging |

## Antibodies

| | |
|---|---|
| Antibodies used | anti-CD24-FITC (#11-0242-85), anti-ScaI-PE (#12-5981-82), and anti-CD140b-biotin (#13-1402-82, eBioscience, San Diego, CA, USA); anti-EpCAM-FITC (#118208), anti-CD61-APC (#104316), and anti-CD29-PE (#102207, BioLegend, San Diego, CA, USA); anti-CD49f-PE/Cy5 (#551129, BD Pharmingen, San Diego, CA, USA); mouse epithelial cell enrichment cocktail (1:20, mixture of biotinylated antibodies against CD45, TER119, BP-1, and CD31; STEMCELL Technologies, Vancouver, Canada); PE-CF594-streptavidin (#562284, BD Biosciences, San Jose, CA, USA); anti-keratin5 (Covance, Denver, PA, USA); anti-E-cadherin (#610181, BD Transduction Laboratories, Lexington, KY, USA); anti-milk specific protein, mouse (RAM/MSP, Nordic-MUbio, Susteren, the Netherlands); anti-Cyclin D1 (ab134175, Abcam, Cambridge, MA, USA);anti-IkBa (#9242S), anti-p-IkBa (#9246L), anti-p100/p52 (#4882S), anti-RelB (#4922S), anti-RelA (#4764S), anti-HDAC2 (#2540S), anti-p-RB (#8516), anti-p-ATK (#4060), anti-ATK (#4691) and Signal Stain Boost IHC detection reagent (#8114P, Cell Signaling Technology, Danvers, MA, USA); anti-Tubulin (CP06, Millipore, Darmstadt, Germany); anti-TRAF6 (sc-7221), and anti-PARP-1 (sc-25780, Santa Cruz Biotechnology, Santa Cruz, CA, USA); and anti-TRAF6 (Ab33915) and anti-RB (Ab181616, Abcam). |
| Validation | Species and applications for all antibodies were described in manufacturer's data sheets. |

## Eukaryotic cell lines

Policy information about cell lines

| | |
|---|---|
| Cell line source(s) | NMuMG cells were kindly provided by Dr. Keiji Miyazawa and Dr. Masao Saitoh in University of Yamanashi. |
| Authentication | NMuMG cells were used for our experiments without cell line authentication. |
| Mycoplasma contamination | Not tested for mycoplasma contamination. |
| Commonly misidentified lines (See ICLAC register) | n/a |

## Animals and other organisms

Policy information about studies involving animals; ARRIVE guidelines recommended for reporting animal research

| | |
|---|---|
| Laboratory animals | 7- to 14-day-old female TRAF6-/-, TRAF6-/+ or wild type on BALB/c background mice were used for transplantation assay as |

| | |
|---|---|
| Laboratory animals | donor mice and FACS analysis. 3-weeks-old female BALB/c mice were used as recipient mice. Recipient mice were analyzed at 8- to 12-weeks-old. |
| Wild animals | n/a |
| Field-collected samples | n/a |
| Ethics oversight | All the mice were maintained under specific pathogen-free conditions and were handled in accordance with the Guidelines for Animal Experiments of the Institute of Medical Science, The University of Tokyo (Tokyo, Japan). |

Note that full information on the approval of the study protocol must also be provided in the manuscript.

# Flow Cytometry

## Plots

Confirm that:

☒ The axis labels state the marker and fluorochrome used (e.g. CD4-FITC).

☒ The axis scales are clearly visible. Include numbers along axes only for bottom left plot of group (a 'group' is an analysis of identical markers).

☒ All plots are contour plots with outliers or pseudocolor plots.

☒ A numerical value for number of cells or percentage (with statistics) is provided.

## Methodology

| | |
|---|---|
| Sample preparation | Mammary fat pads were mechanically dissociated using a scissors and scalpel and then digested for 60 min at 37 °C in DMEM/F12 (Wako) supplemented with 5% FBS, 3 mg/mL collagenase, and 50 microg/mL hyaluronidase. The resulting suspension was sequentially resuspended in 0.25% trypsin- ethylenediaminetetraacetic acid (EDTA) for 5 min, and then in 5 U/mL dispase with 0.1 mg/mL DNase I for 5 min, followed by filtration through a 40-µm mesh. To separate hematopoietic cells, red blood cells, endothelial cells, and fibroblasts, single cells were stained with a mouse epithelial enrichment cocktail (a mixture of biotinylated antibodies against CD45, TER119, BP-1, and CD31) and anti-CD140b-biotin (1:100) for 15 min at 4 °C in antibody staining buffer (HANKs buffer supplemented with 1% FBS). To analyze mammary epithelial cell population in virgin mammary glands, cells were stained with anti-CD24- fluorescein isothiocyanate (FITC) (1:100), anti-CD49f-PE/Cy5 (1:100), anti-CD61-APC (1:100), anti-ScaI-PE (1:100) antibodies, and streptavidin-PE-CF594 (1:100) for 30 min at 4 °C in antibody staining buffer. To analyze the mammary epithelial cell population in P14 and L1 mammary glands, cells were stained with anti-EpCAM-FITC (1:100), anti-CD49f-PECy5 (1:100) antibodies and PE-CF594-streptavidin (1:100) for 30 min at 4 °C in antibody staining buffer. Dead cells were stained with 0.5 microg/mL 7-aminoactinomycin D (7AAD). For cell cycle analysis by quantification of DNA content, sorted primary or NMuMG cells were incubated with Vybrant DyeCycle Green Stain (Thermo Fisher Scientific), a cell-permeable DNA dye, for 30 min at 37 ° C. |
| Instrument | Epics XL flow cytometer (Beckman Coulter, Brea, CA, USA), FACSVerse flow cytometer and FACSAria flow cytometer (BD Biosciences). |
| Software | EXPO32, FACSuite, FACSDiva and FlowJo |
| Cell population abundance | High purity (>95%) of all samples were confirmed by re-analyzing by FACS after sorting. |
| Gating strategy | Doublets (<20%) were distinguished from single cells by plotting FSC height vs FCS area and SSC height vs SCS area. Non-epithelial cells (80%) in single cells were distinguished from epithelial cells by staining with antibodies against CD45, TER119, BP-1, CD31, CD140b. Dead cells (<5%) were distinguished from live cells by staining with 7-aminoactinomycin D (7AAD). Luminal and basal cells in live epithelial population were analyzed by staining with antibodies against CD24, EpCAM and CD49f. For cell cycle analysis, single live cells were analyzed by staining with Vybrant DyeCycle Green Stain. |

☒ Tick this box to confirm that a figure exemplifying the gating strategy is provided in the Supplementary Information.
